# A Versatile Broadband Attached Proton Test Experiment for Routine ^13^C Nuclear Magnetic Resonance Spectroscopy

**DOI:** 10.3390/molecules29040809

**Published:** 2024-02-09

**Authors:** Peter Bigler, Ilche Gjuroski, Dib Chakif, Julien Furrer

**Affiliations:** Department of Chemistry, Biochemistry and Pharmaceutical Sciences, University of Bern, Freiestrasse 3, 3012 Bern, Switzerland; ilche.gjuroski@unibe.ch (I.G.); dib.chakif@unibe.ch (D.C.)

**Keywords:** NMR, ^1^H, ^13^C, APT, broadband APT, ^1^*J_CH_*-tolerance, Cq-only

## Abstract

The proposed broadband attached proton test sequence allows the user to easily record ^13^C nuclear magnetic resonance multiplicity-edited and quaternary-carbon-only spectra. Compared to earlier attached proton test experiments, it preserves both a tolerance for wide ranges of one-bond-coupling constant values and the effective suppression of residual CHn signals in the quaternary-carbon-only spectra. The recording of edited spectra or quaternary-carbon-only spectra is made easy by a single, user-controllable constant. These attributes make the broadband attached proton test experiment attractive for the ^13^C analysis of small molecules, including spectral editing, particularly in high-throughput analysis laboratories.

## 1. Introduction

In ^13^C nuclear magnetic resonance (NMR) spectra, the so-called multiplicity, i.e., the number, n, of magnetically equivalent nuclei, I, bound to spin, S (InS group), is of special interest. Multiplicity provides valuable information to unequivocally find the signals of Cq-, CH-, CH_2_- and CH_3_ -groups and thus to obtain the number of the different carbon types for the molecule under study. Correspondingly designed 1D ^13^C-NMR experiments such as the attached proton test (APT) [1,2], insensitive nuclei enhancement by polarization transfer (INEPT) [3], or distortionless enhancement by polarization transfer (DEPT) [4] were introduced many years ago for this purpose.

The option for multiplicity discrimination has also been incorporated into 2D experiments such as 2D heteronuclear single quantum coherence (HSQC) [5,6] and 3D protein NMR experiments [7]. However, 1D ^13^C- DEPT or -APT are still the most common experiments for assigning ^13^C signals and elucidating molecular structures at a routine level. 

The APT experiment belongs to the family of spin-echo (SE)-based experiments, such as spin-echo Fourier transform (SEFT) [8,9,10], and subspectral editing using a multiple-quantum trap (SEMUT) [11], which exploits heteronuclear Overhauser enhancement to improve sensitivity. Due to their simplicity, these experiments are very robust and are therefore ideal for routine spectroscopy and for educational purposes to introduce the concept of pulse sequences to undergraduate students. The added sensitivity gain achieved with polarization transfer experiments such as DEPT or INEPT has almost stopped the further development of APT-related experiments. However, the simplicity of the APT sequences, and in particular the ability to obtain all the carbon multiplicities, including Cq, is an important advantage that is otherwise only achieved with the polarization enhancement nurtured during attached nucleus testing (PENDANT) [12,13] and the DEPTQ sequences [14,15,16]. Furthermore, and with respect to other applications such as the study of perfluorinated compounds CF_n_, the APT experiment is easier to use than DEPT. Although powerful decoupling methods are essential due to the large ^19^F shift range, the selection and difficult calibration of broadband ^19^F pulses needed for DEPT are no longer necessary.

Recently, we have shown that the “old-fashioned” APT experiment produces similar or sometimes even slightly better results than INEPT or DEPT. [17] We concluded that none of the five experiments tested (APT, DEPT, DEPTQ, Refocused-INEPT, and PENDANT) can be considered an “ultimate” or superior experiment that consistently provides the best signal-to-noise ratio for all ^13^C signals [17].

For the APT experiment, the multiplicity discrimination of carbon signals depends on the length of the delay Δ, which is set to 1/^1^*J*_CH_. This setting enables partial multiplicity editing with positive Cq and CH_2_ signals and negative CH and CH_3_ signals, respectively. With Δ set to 0.5/^1^*J*_CH_, only Cq signals will be detected (Cq-only spectrum). Therefore, with both spectra, the signals of Cq and CH_2_ can be identified unequivocally, while a differentiation between CH and CH_3_ is not possible. 

A long-standing and recurring problem with the APT sequence is that it suffers from low tolerance over wide ranges of ^1^*J*_CH_. For molecules with a wide range of ^1^*J*_CH_ couplings, this affects (i) the sensitivity of the APT spectra in general, with the cancellation of signals or, in the worst case, signals showing the wrong multiplicity, and (ii) a mostly poor suppression of residual CHn signals in the Cq-only spectrum. 

An APT editing method called error self-compensation reached by tau scrambling (ESCORT) has been proposed, which works very well for usual ranges of coupling constants. Yet, ESCORT requires the measurement of four spectra that must then undergo extensive spectral editing, which is not particularly suitable for routine measurements [18].

The CRISIS-APT (CRAPT) experiment, an improved APT with compensation for variations in ^1^*J*_CH_ using ^13^C compensation for refocusing inefficiency with synchronized inversion sweeps (CRISIS) pulses [19], has also been proposed [20]. However, the CRAPT experiment relies on the linear relationship between ^1^*J*_CH_ coupling constant values and carbon chemical shift in optimizing the sweep rate of the adiabatic pulses [21,22,23]. It has, therefore, a low ^1^*J*_CH_ tolerance for chemical functionalities that lie outside this linear region. Furthermore, the CRAPT experiment inadequately suppresses the remaining CHn signals in the Cq-only spectrum.

Finally, more sophisticated APT sequences (compensated attached proton test, CAPT2 and CAPT3) were developed by the McClung’s group to improve the ^1^*J*_CH_ tolerance of APT [24]. The CAPT sequences are more complex, contain a bilinear rotation decoupling (BIRD)*^r,X^* element [25,26] in the center of the preparation period, and are almost identical to the SEMUT-GL sequence, developed for other purposes [27]. 

In this report, we propose a new APT sequence, the broadband attached proton test sequence (BAPT), for measuring partially edited CH_n_ spectra or Cq-only spectra. For wide ranges of ^1^*J*_CH_ coupling constant values, the BAPT CH_n_ spectra show improved ^1^*J*_CH_ tolerance and therefore a higher sensitivity compared to the CAPT sequences. The BAPT Cq-only spectra are characterized by an efficient suppression of the CH_n_ signals with a comparable quality to the spectra of ESCORT, SEMUT-GL, and improved quaternary carbon detection (*i*QCD) sequences [11,27,28], which are known to provide the best Cq-only spectra. Importantly, the present paper aims to improve a single (routine) experiment (APT), i.e., explicitly to not evaluate the tactics of which combination of 1D and/or 2D methods is useful for structure elucidation/verification depending on the structural problem [29,30]. It is obvious, then, when working with sample-limited natural products that it is more difficult to acquire a good-quality ^13^C NMR spectrum than it is to acquire a set of 2D spectra [31].

## 2. Results and Discussion

### 2.1. The Broadband Attached Proton Test Experiment

**Figure 1:**
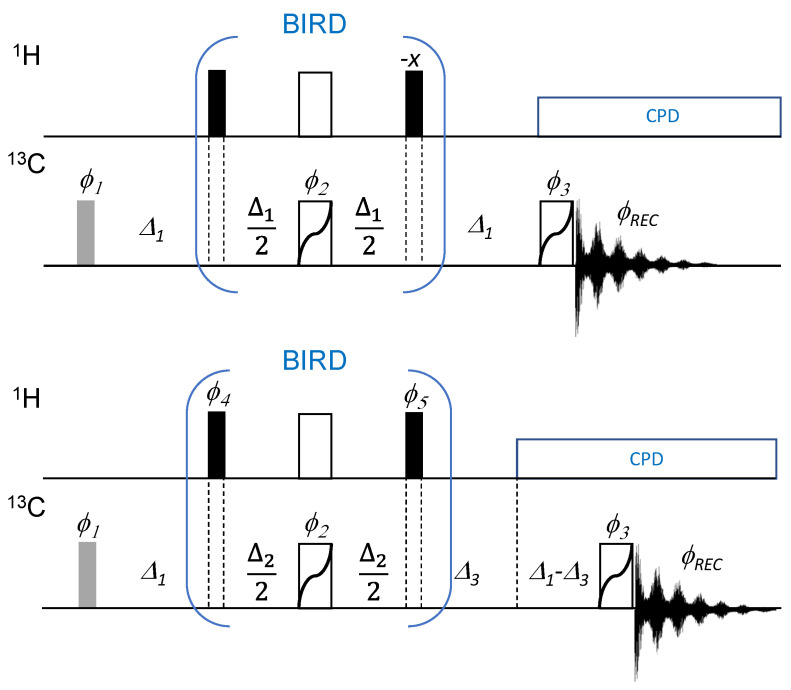
Pulse sequence of the CAPT3 (APTjc in the Bruker pulse program library, top) and the proposed broadband APT (BAPT, bottom) experiments. Thin bars stand for 90° pulses, thick bars for 180° pulses. All ^13^C 180° pulses can be replaced by broadband refocusing pulses [34,35]. The first ^13^C pulse may be set shorter than 90° to allow for a faster repetition rate (shown in grey). In the APTjc experiment, Δ_1_ is set to an average value 1/(2^1^*J*^1^_CH_) [24]. In the BAPT experiment, Δ_1_, Δ_2_, and Δ_3_ are set as described in the text, with Δ_2_ = 1/*J*^2^_CH_ to record APT-like spectra and Δ_2_ = 1/(2*J^2^_CH_) to record Cq-only spectra. The following phase cycling is applied: *φ*_1_ = 4(*x*), 4(−*x*), 4(*y*), 4(−*y*), *φ*_2_ = *x*, *y*, −*x*, −*y*, *y*, −*x*, −*y*, *x*, −*x*, −*y*, *x*, *y*, −*y*, *x*, *y*, −*x*, *φ*_3_ = *x*, *y*, *y*, *x*,( *y*, *x*, *x*, *y*)_2_, *x*, *y*, *y*, *x*, *φ_REC_* = *x*, *x*, −*x*, −*x*, *y*, *y*, −*y*, −*y*. BAPT experiment: *φ*_4_ = *x* (APT-like mode); *x*, *x*, −*x*, −*x* (Cq-only mode), *φ*_5_ = −*x* (APT mode); *y*, −*y* (Cq-only mode). Phases not shown are applied along the *x*-axis.

The broadband APT (BAPT) pulse sequence differs from the recommended CAPT3 pulse sequence [24] as follows (Figure 1): (i) Instead of a delay, Δ_1_, set to one single ^1^*J*_CH_ value, three delays, Δ_1_, Δ_2_, and Δ_3_, are used and set for three estimated one-bond coupling constants, ^1^*J*^1^_CH_, ^1^*J*^2^_CH_, and ^1^*J*^3^_CH_, a long-known strategy that has been implemented in low-pass *J* filters [32], in SEMUT-GL [27], in HMBC experiments [33], and, recently, in DEPTQ+ experiments [16]. (ii) Like the basic APT experiment, the BAPT experiment can be used to provide either an APT spectrum or a Cq-only spectrum. For an APT spectrum, Δ_1_ = 1/(2*^1^*J*^1^_CH_), Δ_2_ = 1/(^1^*J*^2^_CH_), and Δ_3_ = 1/(2*^1^*J*^3^_CH_). To measure a Cq-only spectrum, the delay, Δ_2_, must be halved, i.e., (1/(^1^*J*^2^_CH_) → 1/(2*^1^*J*^2^_CH_)), and the phase cycle for the two proton 90° pulses (see Figure 1) must be redefined. For routine NMR spectroscopy, the choice between an APT or a Cq-only spectrum is made very simply and conveniently using just the value of a constant (1 = APT; 2 = Cq-only). The delay, Δ_2_, and the phase programs for the two proton 90° pulses are adjusted accordingly and automatically (Supplementary Information).

### 2.2. Analysis of the Broadband Attached Proton Test Experiment

#### 2.2.1. Attached Proton Test Mode

The CAPT3 and BAPT sequences with the phases and delays adjusted to generate an APT spectrum were first evaluated. In Table 1, the observable in-phase ^13^C coherences for Cq, CH, CH_2_, and CH_3_ spin systems present just before acquisition are summarized. In the rest of the manuscript, for convenience, we will use the name of the pulse program, APTjc, to discuss the CAPT2 and CAPT3 sequences. As for the coupling between a carbon and its directly bound protons, ^1^*J*_CH_ = *J* is the value of the actual coupling and ^1^*J*^1^_CH_, ^1^*J*^2^_CH_, ^1^*J*^3^_CH_ are the values of the couplings used to define delays Δ_1_, Δ_2_, and Δ_3_, respectively. 

As shown in Table 1, the terms shown in bold only occurred when the delays were not optimally adjusted, i.e., Δ*_i_*≠ 1/(2*J_CH_) [24,27]. The intensities of all CHn groups were primarily determined by the respective terms 1 and 2, with term 1 dominating, except for CH groups and large mismatches between *J* and Δ*_i_* [24]. For CH_2_ groups, simple numerical evaluations show that term 3 stayed small for their full range of coupling constants (~110–185 Hz) and can be neglected. For CH_3_ groups, terms 3 and 4 remained small for their full range of coupling constants (~90–140 Hz) and can be neglected as well.

From the expressions provided in Table 1, several conclusions and recommendations can be derived for the best adjustment of the delays Δ_1_–Δ_3_ in the BAPT experiment:

In the APTjc experiment, with the delay Δ_1_ set to Δ_1_ = 1/(2*^1^*J*_CH_), an excellent tolerance over a wide range of one-bond *J* coupling constants could be achieved [24]. However, we found that an even better ^1^*J*_CH_ tolerance could be achieved by setting Δ_1_ in the BIRD sandwich to Δ_1_ = 1/(^1^*J*^1^_CH_) (Figure S2). That is why Δ_2_ is defined as 1/(^1^*J^2^*_CH_) in the BIRD sandwich of the BAPT experiment for recording an APT spectrum. 

#### 2.2.2. Standard Range of One-Bond J_CH_ Coupling Constants (110–175 Hz)

**Figure 2:**
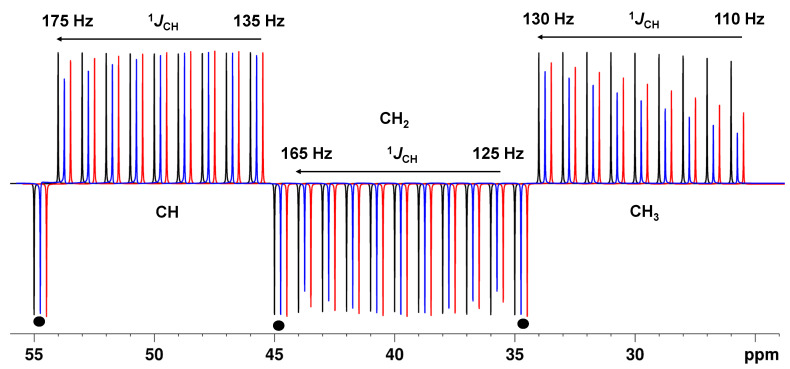
Theoretical intensity of CH, CH_2_, and CH_3_ groups as a function of the ^1^*J*_CH_ value for the APT pulse sequence, Δ_1_ = 1/(^1^*J*^1^_CH_) (**--**), the APTjc pulse sequence, Δ_1_ = 1/(2*^1^*J*^1^_CH_) (**--**), and the BAPT pulse sequence, Δ_1_ = 1/(2*^1^*J*^1^_CH_), Δ_2_ = 1/^1^*J*^2^_CH_, and Δ_3_ = 1/(2*^1^*J*^3^_CH_) (**--**). For APT and APTjc, Δ_1_ was set to match a coupling constant value of 145 Hz. For BAPT, Δ_1_ and Δ_3_ were set to match a coupling constant value of 130 Hz, and Δ_2_ was set to match a coupling constant value of 175 Hz. Three quaternary carbons at 35, 45, and 55 ppm have been added for comparison and are shown with ●. For CH groups, the ^1^*J*_CH_ value was varied from 135 to 175 Hz (right to left) in steps of 5 Hz. For CH_2_ groups, the ^1^*J*_CH_ value was varied from 125 to 165 Hz (right to left) in steps of 5 Hz. For CH_3_ groups, the ^1^*J*_CH_ value was varied from 110 to 130 Hz (right to left) in steps of 2.5 Hz. Full relaxation was considered. Simulations were performed using NMRSIM for Windows.

Simple calculations and simulations (Figures S3–S5) show that for an APT-type spectrum with the BAPT experiment, the delay Δ_2_ should be adjusted for all carbon types for large coupling constant values, typically 160–170 Hz, to obtain the best ^1^*J*_CH_ tolerances. In contrast, the delays Δ_1_ and Δ_3_ should be optimized for the typical ^1^*J*_CH_ ranges of CH_2_ and CH_3_ groups, and thus a value around 130 Hz provided the best results (Figure 2 and Figures S3–S5). With these optimizations, the intensity of CH groups was at a maximum (>99%) and constant over the coupling constant range of 130–175 Hz (Figure 2 and Figure S3). For the APTjc sequence, with Δ_1_ adjusted for an average coupling constant of 145 Hz, high intensities (>98%) were obtained for aliphatic CH groups, while the intensity could drop to ~90% for aromatic carbons with ^1^*J*_CH_ coupling constants of ~175 Hz. 

For the BAPT experiment, excellent tolerances and intensities close to the theoretical maximum were also obtained for CH_2_ (>96%) and particularly for CH_3_ groups (>90%) over their typical ^1^J_CH_ ranges (125–165 Hz, CH_2_; 110–135 Hz, CH_3_). For the APTjc sequence, the intensity dropped to 89% (CH_2_ groups) and a low 53% (CH_3_ groups) with Δ_1_ adjusted for an average coupling constant of 145 Hz (Figure 2, Figures S4 and S5). As expected, both the APTjc and the BAPT sequence were superior to the APT sequence in every respect, i.e., in terms of overall sensitivity and tolerance to *J*-mismatch, especially for CH_3_ groups.

We also simulated the corresponding spectra for a hypothetical molecule and included spectral noise in the simulations to mimic the experimental data as closely as possible (Figure S6). The spectra confirmed the slight superiority of the BAPT sequence compared to the APTjc sequence: The improved *J*-tolerance achieved with BAPT with only a weak *J* dependance was most impressive for CH_3_ groups, while almost no *J*-dependance could be seen for the aliphatic CH_2_ and CH groups (Figure 2). 

#### 2.2.3. Full Range of One-Bond J_CH_ Coupling Constants (110–250 Hz)

**Figure 3:**
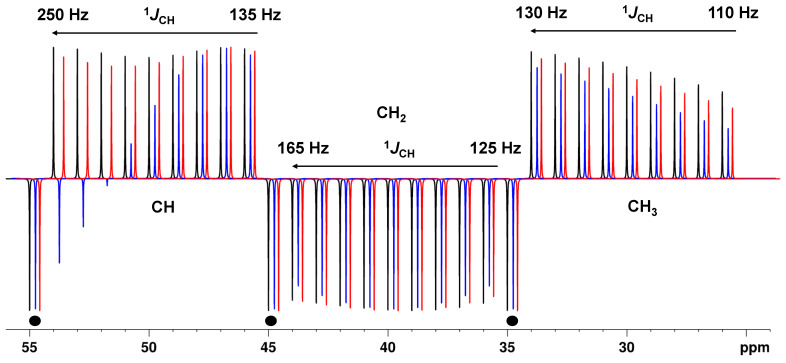
Theoretical intensity of CH, CH_2_, and CH_3_ groups as a function of the ^1^*J*_CH_ value for the APT pulse sequence, Δ_1_ = 1/(^1^*J*^1^_CH_) (**--**), the APTjc pulse sequence, Δ_1_ = 1/(2*^1^*J*^1^_CH_) (**--**), and the BAPT pulse sequence, Δ_1_ = 1/(2*^1^*J*^1^_CH_), Δ_2_ = 1/^1^*J*^2^_CH_, and Δ_3_ = 1/(2*^1^*J*^3^_CH_) (**--**). For APT and APTjc, Δ_1_ was set to match a coupling constant value of 145 Hz. For BAPT, Δ_1_ and Δ_3_ were set to match a coupling constant value of 145 Hz, and Δ_2_ was set to match a coupling constant value of 250 Hz. Three quaternary carbons at 35, 45, and 55 ppm have been added for comparison and are shown with ●. For CH groups, the ^1^*J*_CH_ value was varied from 135 to 250 Hz (right to left) in steps of 15 Hz. For CH_2_ groups, the ^1^*J*_CH_ value was varied from 125 to 165 Hz (right to left) in steps of 5 Hz. For CH_3_ groups, the ^1^*J*_CH_ value was varied from 110 to 130 Hz (right to left) in steps of 2.5 Hz. Full relaxation was considered. Simulations were performed using NMRSIM for Windows.

When the full range of one-bond *J*_CH_ coupling constants (110–250 Hz) is considered, the same conclusions apply for the delay Δ_2_: The calculations and simulations show that for the BAPT experiment, the delay Δ_2_ must also be adjusted to very large coupling constant values, in this case, typically to 240–250 Hz. For the delays Δ_1_ and Δ_3_, we found that the best compromise was achieved when both delays were adjusted close to the standard average value of 145 Hz (Figure 3 and Figures S7–S9). With these optimized delays, Δ*_i_*, an astonishingly high and uniform amplitude for all sizes of coupling constants (130–250 Hz) in CH groups was obtained (>95%) (Figure 3 and Figure S7). For the APTjc sequence, the intensity was also high and uniform, but it could drop to ~85%. 

For the CH_2_ and CH_3_ groups, the BAPT sequence showed a slightly superior sensitivity compared to the APTjc sequence (Figure 3, Figures S8 and S9). Both BAPT and the APTjc sequences were again superior to the APT sequence for all carbon types, particularly for the alkyne group, for which the APT sequence produced an intense resonance with the wrong sign (Figure 3).

#### 2.2.4. Cq-Only Mode

The APTjc experiment is intended exclusively for measuring APT spectra. However, after a minor modification (Figure S1), it can also be used to measure Cq-only spectra Therefore, we evaluated the performance of this modified APTjc and the BAPT sequence with its phases and delays adjusted to provide Cq-only spectra. In Table 2, the residual in-phase ^13^C coherences for CH, CH_2_, and CH_3_ spin systems present just before acquisition are summarized. Note that identical expressions to those derived for the BAPT experiment were obtained for the SEMUT-GL experiment, which is known to provide the best Cq-only spectra [27].

#### 2.2.5. Standard Range of One-Bond J_CH_ Coupling Constants (110–175 Hz)

**Figure 4:**
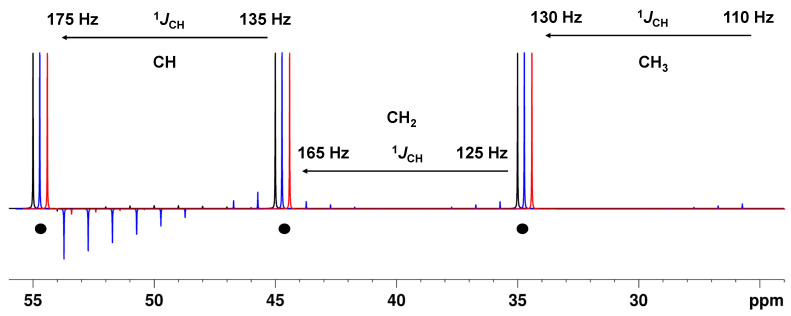
Theoretical residual intensity of CH, CH_2_, and CH_3_ groups in a Cq-only spectrum as a function of the ^1^*J*_CH_ value for the APT pulse sequence, Δ_1_ = 1/(2*^1^*J*^1^_CH_) (**--**), the modified APTjc pulse sequence, Δ_1_ = 1/(2*^1^*J*^1^_CH_) (**--**), and the BAPT pulse sequence, Δ_1_ = 1/(2*^1^*J*^1^_CH_), Δ_2_ = 1/(2*^1^*J*^2^_CH_), and Δ_3_ = 1/(2*^1^*J*^3^_CH_) (**--**). The spin system and all other parameters were identical to those used for Figure 2.

Using the same three ^1^*J*^i^_CH_ values for the delays Δ_1_, Δ_2_, and Δ_3_ as recommended to obtain an APT spectrum but with Δ_2_ redefined accordingly—Δ_2_ = 1/(2*^1^*J*^2^_CH_)—to measure a Cq-only spectrum, the following results were obtained:

The modified APTjc and BAPT sequences were superior to the APT sequence in every respect: for the CH groups, which are the most critical in this regard, the residual signals are expected to be below the noise level but clearly visible in the Cq-only spectrum recorded with the APT sequence (Figure 4, Figures S10 and S11). According to the expressions shown in Table 2, the residual signals of the aliphatic CH_2_ and CH_3_ groups, which are easier to suppress, are expected to be below the noise level for the APTjc and BAPT pulse sequences but could potentially be visible in the Cq-only spectrum recorded with the APT sequence (Figure 4 and Figure S11).

#### 2.2.6. Full Range of One-Bond J_CH_ Coupling Constants (110–250 Hz)

**Figure 5:**
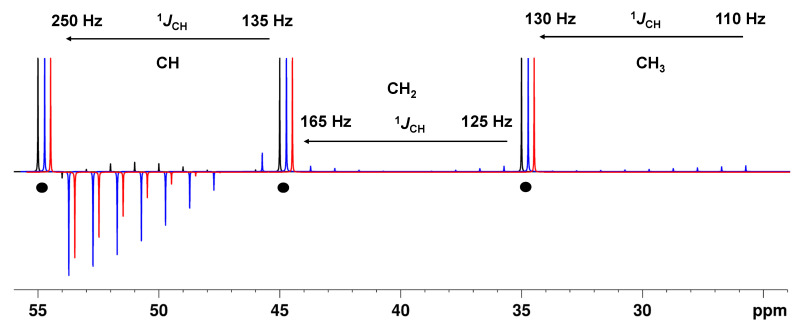
Theoretical residual intensity of CH, CH_2_, and CH_3_ groups in a Cq-only spectrum as a function of the ^1^*J*_CH_ value for the APT pulse sequence (**--**), the APTjc pulse sequence, Δ_1_ = 1/(2*^1^*J*^1^_CH_) (**--**), and the BAPT pulse sequence, Δ_1_ = 1/(2*^1^*J*^1^_CH_), Δ_2_ = 1/(2*^1^*J*^2^_CH_), and Δ_3_ = 1/(2*^1^*J*^3^_CH_) (**--**). The spin system and all other parameters were identical to those used for Figure 3.

For the full range of coupling constants (110–250 Hz), the BAPT sequence was also clearly superior to the APT and APTjc sequences in every respect: the intensities of CH artifacts were very weak (alkyne CH group) or remained below the noise level (aromatic CH group), as expected, while intense residual signals of these CH groups are potentially visible in the Cq-only spectra recorded with the APT and APTjc experiment (Figure 5, Figures S12 and S13). The residual signals of the less critical aliphatic CH_2_ and CH_3_ groups are also expected to be below the noise level for the APTjc and BAPT pulse sequences but could potentially be visible in the Cq-only spectrum recorded with the APT sequence (Figure 5 and Figure S13).

## 3. Experimental Data

### 3.1. Cholesteryl Acetate

#### 3.1.1. Attached Proton Test Spectrum

**Figure 6:**
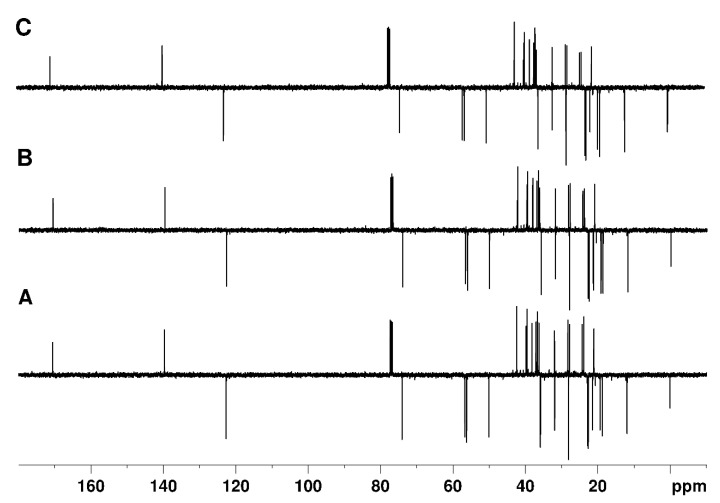
APT (**A**), APTjc (**B**), and BAPT (**C**) spectra of cholesteryl acetate. For APT and APTjc, the delay, Δ, was adjusted to a coupling constant of 145 Hz, while for BAPT, Δ_1_ and Δ_3_ were adjusted to a coupling constant of 130 Hz, and Δ_2_ was adjusted to a coupling constant of 170 Hz.

**Figure 7:**
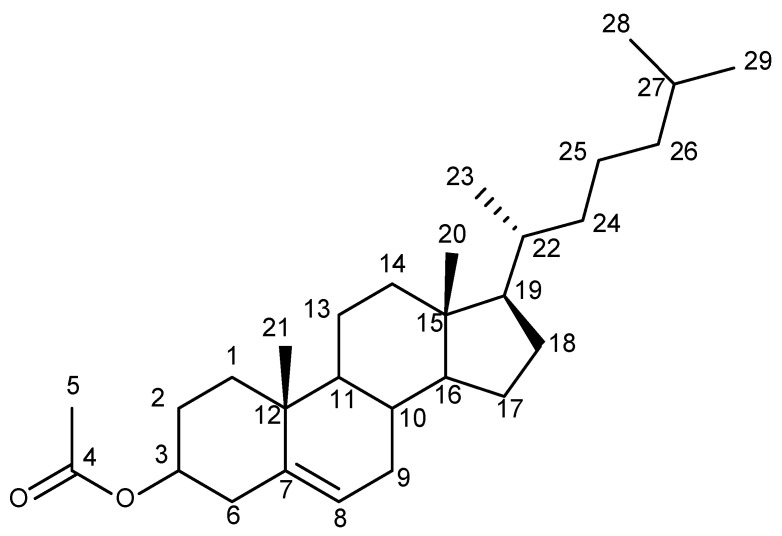
Molecular structure of cholesteryl acetate and atom numbering used in the text.

**Figure 8:**
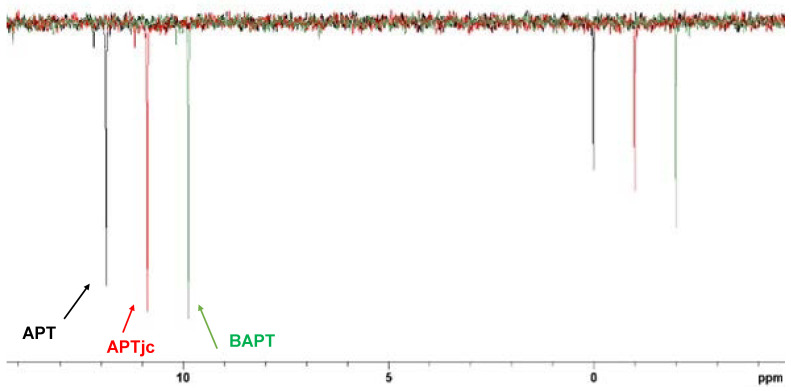
Resonances of C12 (δ = 12 ppm, ^1^*J*_CH_ = 122 Hz) of cholesteryl acetate and of TMS (δ = 0 ppm, ^1^*J*_CH_ = 117 Hz) dissolved in 0.7 mL of CDCl_3_ recorded with the APT (black), APTjc (red), and BAPT (green) experiments.

In Figure 6, the APT, APTjc, and BAPT spectra of 100 mmol cholesteryl acetate (Figure 7) dissolved in CDCl_3_ are shown. For APT and APTjc, the delay Δ_1_ was set to a coupling constant of 145 Hz, while for BAPT, Δ_1_ and Δ_3_ were set to a coupling constant of 130 Hz, and Δ_2_ was set to a coupling constant of 170 Hz, respectively. As expected for a molecule with a narrow range of one-bond coupling constants (120–153 Hz), similar spectra were obtained. However, the intensities of some methyl groups (Me20 at δ = 12 ppm) and of TMS at δ = 0 ppm were slightly more intense in the BAPT spectrum (Figure 8), as predicted, while other resonances (C3 at 74.2 ppm, C8 at 122.8 ppm) were slightly less intense (Figure S14). 

#### 3.1.2. Cq-Only Spectrum

**Figure 9:**
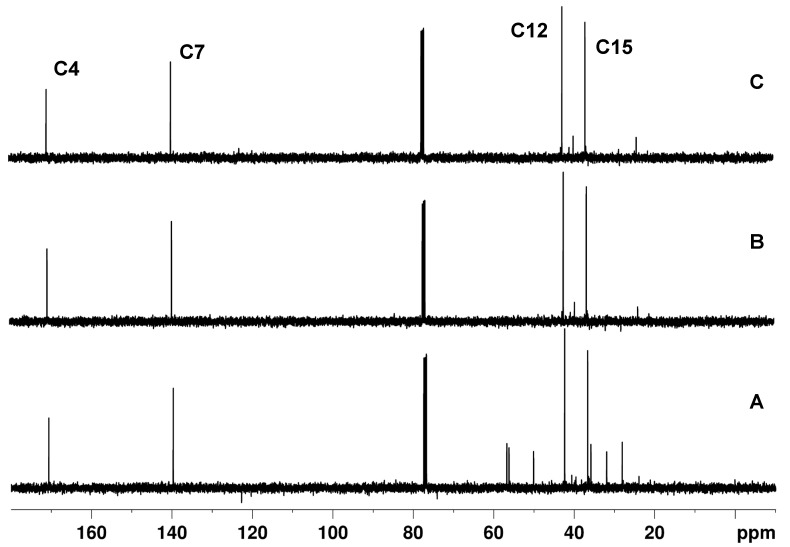
Cq-only spectra of cholesteryl acetate obtained using the APT (**A**), modified APTjc (**B**), and BAPT (**C**) pulse sequences. For APT and APTjc, the delay Δ was adjusted to a coupling constant of 145 Hz, while for BAPT, Δ_1_ and Δ_3_ were adjusted to a coupling constant of 130 Hz, and Δ_2_ was adjusted to a coupling constant of 170 Hz.

In Figure 9, the Cq-only spectra obtained using the APT, the modified APTjc, and the BAPT pulse sequences of cholesteryl acetate are shown. For APT and APTjc, the delay, Δ, was set to a coupling constant of 145 Hz, while for BAPT, Δ_1_ and Δ_3_ were set to a coupling constant of 130 Hz, and Δ_2_ was set to a coupling constant of 170 Hz, respectively. As expected, the spectrum obtained using the APT sequence was marred by numerous, sometimes quite intense, CH and CH_2_ artifacts. The spectra demonstrate the ability of the APTjc and the new BAPT pulse sequences to efficiently remove the signals originating from protonated carbons and to provide clean Cq-only spectra, comparable to those obtained using the SEMUT-GL and *i*QCD sequences, which are known to provide the best-possible Cq-only spectra [27,28].

### 3.2. 4-Methyl-N,N-(prop-2-yn-1-yl)aniline

#### 3.2.1. APT Spectrum

**Figure 10:**
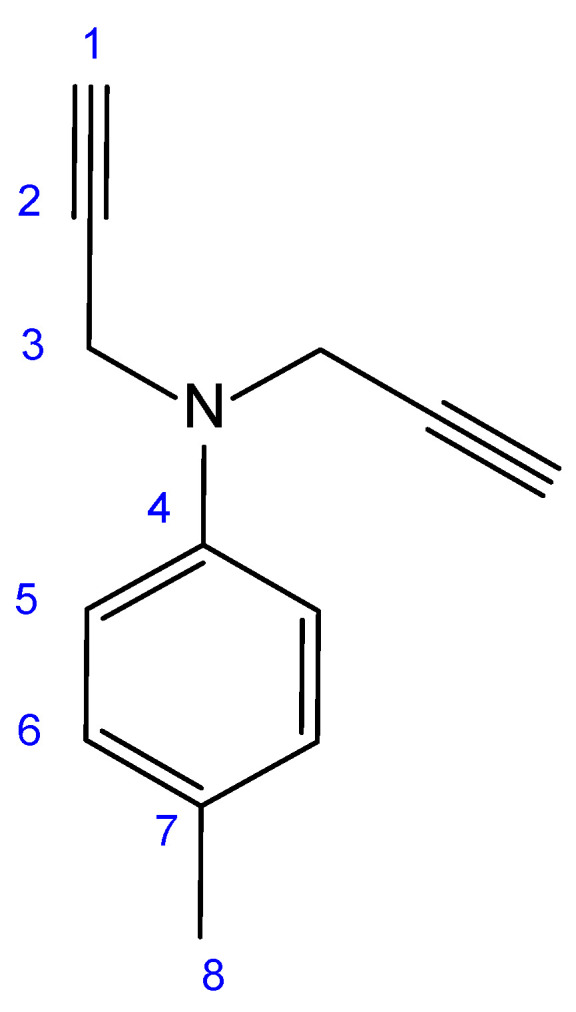
Molecular structure of 4-methyl-*N*,*N*-(prop-2-yn-1-yl)aniline and atom numbering used in the text.

To better evaluate the effective performance of the BAPT experiment in a more challenging situation, we used a sample of ~30 mg of 4-methyl-*N*,*N*-di(prop-2-yn-1-yl)aniline dissolved in 0.7 mL of CDCl_3_ (Figure 10). This molecule has alkane, aromatic, and alkyne groups that cover the entire range of ^1^*J*_CH_ coupling constants (125 Hz for ^1^*J*_C8H8_ to 248 Hz for ^1^*J*_C1H1_). Also, note the special case of C2 at 79.4 ppm, which behaves like a pseudo-quaternary carbon with its large two-bond coupling constant ^2^*J*_C2H1_ of 49 Hz. C2 should therefore be viewed as a CH group with a small ^1^*J*_CH_ coupling constant.

**Figure 11:**
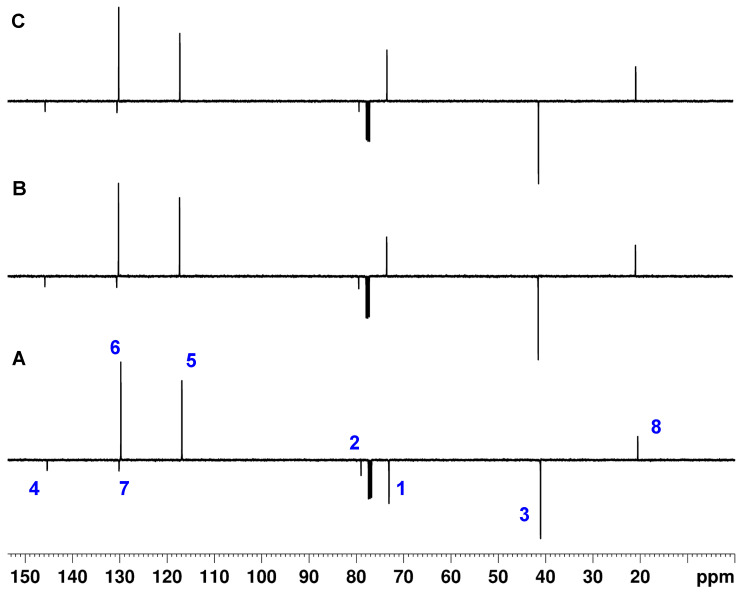
APT (**A**), APTjc (**B**), and BAPT (**C**) spectra of ~10 mg of 4-methyl-*N*,*N*-di(prop-2-yn-1-yl)aniline dissolved in 0.7 mL of CDCl_3_. For APT and APTjc, the delay Δ_1_ was adjusted for a coupling constant ^1^*J*^1^_CH_ of 145 Hz. For BAPT, the delays Δ_1_ and Δ_3_ were adjusted for a coupling constant ^1^*J*^1,3^_CH_ of 145 Hz, and the delay Δ_2_ was adjusted for a coupling constant ^1^*J*^2^_CH_ of 240 Hz. The relaxation delay was 2 s. All other parameters were identical to those described in the Materials and Methods Section.

**Figure 12:**
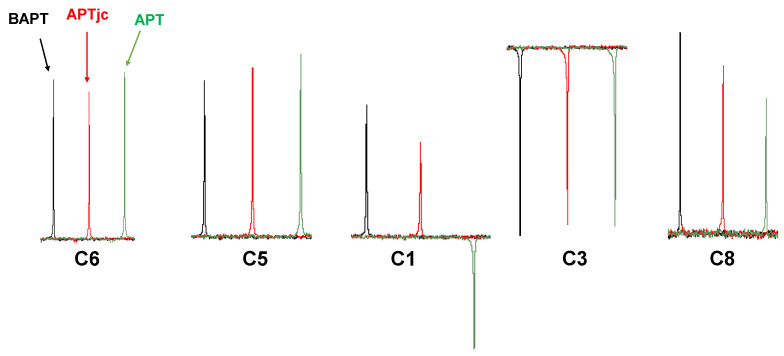
Resonances of C1, C3, C5, C6, and C8 of ~10 mg of 4-methyl-*N*,*N*-di(prop-2-yn-1-yl)aniline dissolved in 0.7 mL of CDCl_3_ recorded with the APT (green), APTjc (red), and BAPT (black) experiments.

The standard APT spectrum of 4-methyl-*N*,*N*-di(prop-2-yn-1-yl)aniline is misleading. On the one hand, the resonance of the C8 methyl group at 20.6 ppm was very weak, and on the other hand, and more seriously, the resonance of C1 at 73.1 ppm appeared with the wrong sign and thus also provided the wrong multiplicity (Figure 11). As has been shown theoretically, these two findings were due to the large discrepancy between the actual ^1^*J*_CH_ coupling constants in the acetylene- (~250 Hz) and methyl (~120 Hz) groups and the average coupling constant used to define the delays in the APT experiment, ^1^*J*^1^_CH_ = 145 Hz. Note that using an average ^1^*J*^1^_CH_ coupling constant of 185 Hz resulted in an APT spectrum with correct signal signs, but the resonance of the C8 methyl group at 20.6 ppm was almost invisible (not shown). In the APTjc spectrum of 4-methyl-*N*,*N*-di(prop-2-yn-1-yl)aniline, all resonances appeared with the correct signs according to their multiplicities, as well as an overall good intensity. In the BAPT spectrum, the signal intensities of C3 and C6 were slightly better, that of C5 slightly worse, while the signals of C1 and C8 were more intense (Figure 12). The BAPT spectrum was recorded according to the theoretical findings (Figure 3 and Figures S7–S9); the delay Δ_2_ was adjusted to a coupling constant of 240 Hz, and the delays Δ_1_ and Δ_3_ were adjusted to a value of 145 Hz. 

The ability to independently optimize the three delays Δ_1_–Δ_3_ offers great flexibility with the BAPT sequence for specific optimization. This makes it possible to specifically increase the intensity of selected resonances and reduce others to achieve a sufficient intensity for all resonances in the shortest possible time and thus optimize the measurement time. As an example, by changing the values of the three delays Δ_1_–Δ_3_, the intensity of the less intense carbons C1 and C8 can be slightly increased at the expense of the more intense carbons C3, C5, and C6 (Figure S15).

#### 3.2.2. Cq-Only Spectrum

**Figure 13:**
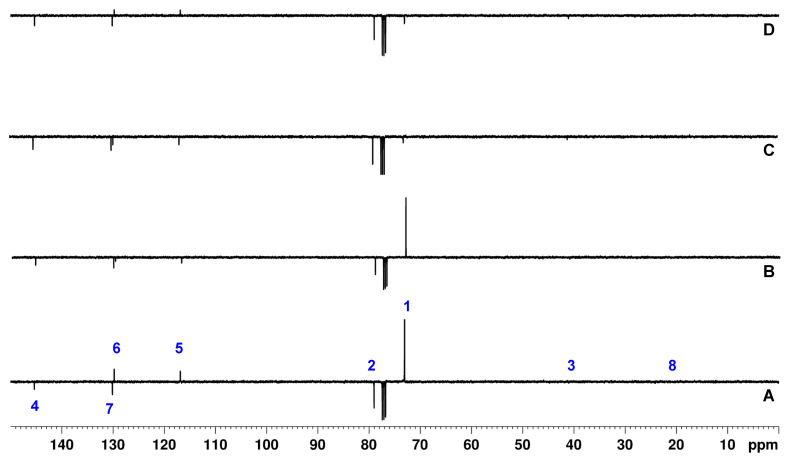
APT (**A**), APTjc (**B**), BAPT (**C**), and SEMUT-GL (**D**) Cq-only spectra of ~10 mg of 4-methyl-*N*,*N*-di(prop-2-yn-1-yl)aniline dissolved in 0.7 mL of CDCl_3_. For the APT and the modified APTjc experiments, the delay Δ_1_ was adjusted for a coupling constant ^1^*J*^1^_CH_ of 145 Hz. For BAPT, the delays Δ_1_ and Δ_3_ were adjusted for a coupling constant ^1^*J*^1,3^_CH_ of 145 Hz, and the delay *Δ_2_* was adjusted for a coupling constant ^1^*J^2^*_CH_ of 240 Hz. For SEMUT-GL, the delay Δ_1_ was adjusted for a coupling constant ^1^*J*^1,3^_CH_ of 133 Hz, the delay Δ_2_ was adjusted for a coupling constant ^1^*J*^2^_CH_ of 235 Hz, and the delay Δ_3_ was adjusted for a coupling constant ^1^*J*^1,3^_CH_ of 191 Hz [27,32]. The tip angle of the first ^13^C pulse was 30°, and the relaxation delay was 4 s. All other parameters were identical to those described in the Materials and Methods Section.

In Figure 13, the Cq-only spectra of 4-methyl-*N*,*N*-di(prop-2-yn-1-yl)aniline recorded with the APT, the modified APTjc, and BAPT sequences are shown. The Cq-only spectra obtained with the modified APTjc and BAPT sequences showed similar suppression degrees for CHn signals, except for a strong artifact of C1 at 73.1 ppm visible in in the APTjc spectrum. As expected, the APT sequence resulted in a significantly worse suppression of the CH_n_ signals. The quality of the Cq-only spectrum obtained with the optimized SEMUT-GL sequence [27,32] was identical to that obtained with the BAPT sequence, except that the sign of the residual CH group signals was reversed due to the use of different Δ_1_–Δ_3_ delays.

Finally, we would like to highlight two situations in which 1D ^13^C BAPT experiments may prove advantageous over the standard heteronuclear inverse 2D ^1^H-^13^C correlation experiments HSQC and HMBC, even though a fair comparison proves tricky, because the philosophies, spectroscopic information, and characteristics of the experiments are so different. 

**Figure 14:**
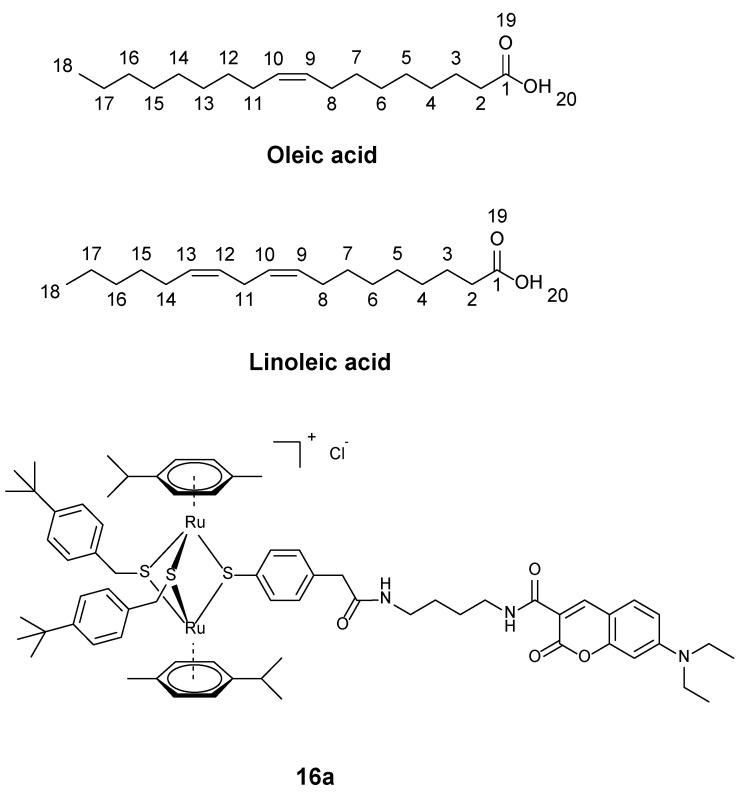
Molecular structures of oleic acid, linoleic acid, and the diruthenium–coumarin conjugate **16a** [36], and atom numbering used in the text.

The first situation arises when analyzing samples that produce very compact ^13^C spectra, including many resonances in a reduced spectral window. This situation can arise in the analysis of mixtures, e.g., fatty acid mixtures, or in the following example, which contains a mixture of one of our coumarin fluorophore-bound diruthenium complexes, compound **16a** [36] (Figure 14), with oleic and linoleic acids.

**Figure 15:**
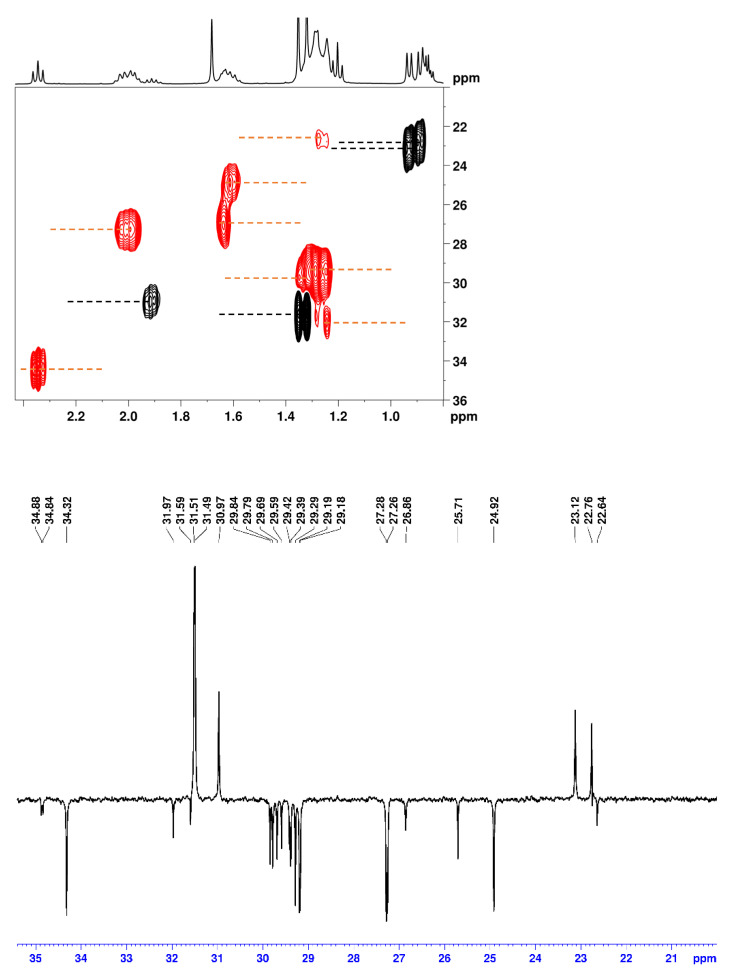
(**Top**): part of the 2D ^1^H-^13^C edited HSQC spectrum of an equimolar (30 mmol) mixture of oleic acid, linoleic acid and compound **16a** dissolved in CDCl_3_. CH and CH_3_ signals are phased up (black peaks) and CH_2_ signals are phased down (red peaks). (**Bottom**): BAPT spectrum of the same mixture, CH and CH_3_ signals are phased up and CH_2_ signals are phased down. The experimental parameters of the BAPT experiment were identical to those used for Figure 6.

For instance, in the 2D ^1^H-^13^C edited HSQC spectrum of this mixture, only 12 carbons can be unambiguously differentiated in the region 20–35 ppm (Figure 15). Actually, there are more than 30 different carbons in this region: the aliphatic CH_2_ carbons **2**–**7** and **12**–**16** (oleic acid), the aliphatic CH_2_ carbons **2**–**7** and **15**–**16** (linoleic acid), the allylic carbons **8** (oleic and linoleic acids), **11** (oleic acid), and **14** (linoleic acid), the bis-allylic carbon **11** (linoleic acid), as well as various CH_2_ and CH_3_ groups of the diruthenium compound **16a** [36]. Those carbons are easily identified in a 1D ^13^C APT or BAPT spectrum due to the much better resolution (Figure 15). It is of course possible to unambiguously identify and assign all carbons using 2D HSQC experiments, but this requires a very high resolution in the indirect dimension (F1, ^13^C), which can take very long measurement times, even if modern techniques such as non-uniform sampling (NUS) are used [37]. An alternative is the recording of band-selective 2D HSQC experiments [38], but these are not always easy to implement and use routinely and in a fully automatic mode.

The same problem occurs with the 2D ^1^H-^13^C HMBC spectrum of this mixture; for instance, only ~20 carbons can be unambiguously differentiated in the region 120–180 ppm (Figure S16). There are 27 different carbons in this region, all easily identified in a 1D ^13^C APT or BAPT spectrum (Figure S16). It is of course possible to unambiguously identify and assign all carbons using the 2D HMBC experiment, but, again, this requires experiments with a very high resolution in the indirect dimension (F1, ^13^C), i.e., a long measurement time or the recording of band-selective HMBC experiments [39].

**Figure 16:**
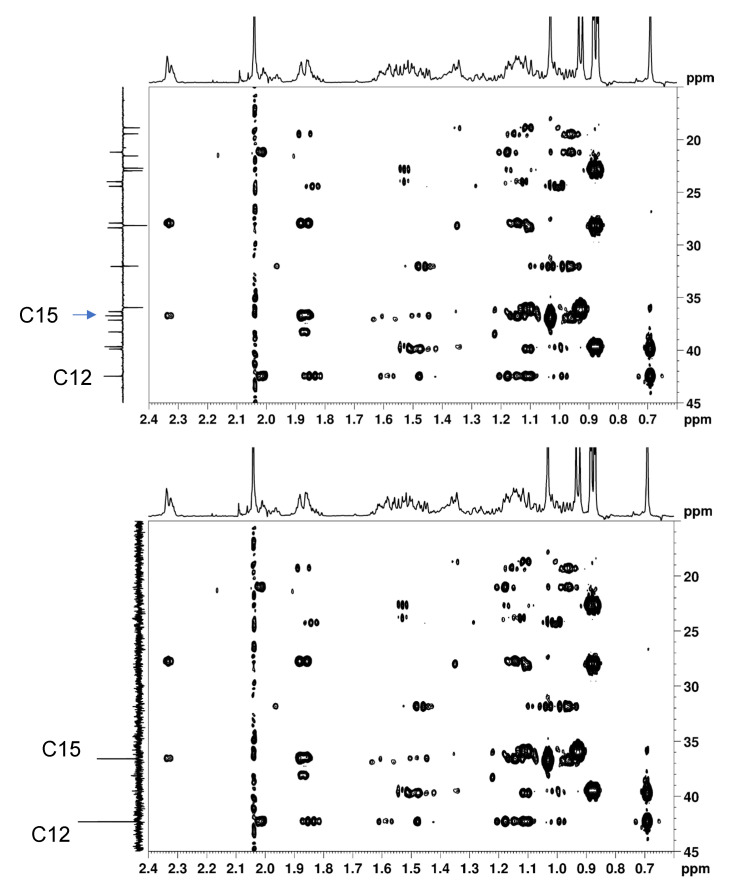
Part of the 7 Hz adjusted HMBC spectrum of cholesteryl acetate. Top: BAPT CHn, Cq spectrum set as external *F*_1_ projection. Bottom: BAPT Cq-only spectrum set as external *F*_1_ projection. The experimental parameters of the BAPT experiments were identical to those used for Figure 9.

Another potentially interesting benefit of 1D BAPT experiments is the ability to record clean Cq-only experiments. Indeed, the resulting Cq-only spectra can be used as F1 projections in HMBC or LR-HSQMBC spectra [40]. As seen in Figure 16, the identification and the correct attribution of the two quaternary carbons of cholesteryl acetate in the aliphatic region (C12 at 42.5 ppm and C15 at 36.7 ppm) are extremely facilitated in the HMBC spectrum when the clean BAPT Cq-only spectrum is set as the F1 projection. The distinction between CH_2_ and Cq resonances in APT spectra is not always trivial. Thus, when using the APT spectrum as an F1 projection, the identification and correct attribution of C12 and C15 are more difficult and require an almost complete analysis and attribution of all the carbons (Figure 16).

## 4. Conclusions

Despite the availability of powerful 2D methods such as HSQC and HMBC, 1D ^13^C NMR experiments are still popular as routine experiments due to their simplicity and usually short measurement time, be it in daily use, e.g., on open-access NMR spectrometers for reaction control, or for the structure verification of smaller molecules. Among them, the APT experiment is remarkably simple and robust, making it ideal for routine spectroscopy in the autosampler mode. However, when measuring CHn spectra, it suffers from a poor tolerance to wide ranges of ^1^*J*_CH_ values and, as a secondary application, from the poor suppression of residual CH_n_ signals in Cq-only spectra.

Given these problems and inspired by modified APT pulse sequences, we have developed the BAPT experiment, which provides a remarkably uncomplicated way to obtain both CHn spectra with an excellent tolerance for wide ranges of ^1^*J*_CH_ values and Cq-only spectra with the efficient suppression of unwanted CH_n_ signals. This improved performance, which has been proven both theoretically and experimentally, is achieved with three different delays Δ_1_, Δ_2_, and Δ_3_ compared to just one in the basic APT experiment. The three delays can be set for three different one-bond coupling constants and—according to a simple recipe—optimally adapted to the sample under investigation. 

The high performance in terms of ^1^*J*_CH_ tolerance and CHn suppression achieved with the dual-purpose BAPT experiment is quite comparable to the highest qualities achieved with the appropriately optimized SEMUT-GL or the single-purpose experiments designed exclusively for the measurement of pure CHn- (APTjc) and pure Cq-only spectra (iQCD). 

Finally, the recording of APT and Cq-only spectra is most effectively accomplished by adjusting a single constant, which controls the delay (∆_2_) and phase programs for the two proton 90° pulses. These features increase the attractiveness of the proposed BAPT experiment for the ^13^C analysis of small molecules, especially in high-throughput analysis laboratories.

## 5. Materials and Methods

### 5.1. NMR Measurements

All experiments were recorded on a Bruker AvanceIIIHD 400 MHz NMR spectrometer equipped with a 5 mm BBFO Smartprobe^®^ direct probehead (Bruker, Billerica, MA, USA). The samples used were (i) 30 mg of cholesteryl acetate dissolved in 0.7 mL of CDCl_3_ (standard test sample provided with Bruker NMR spectrometers), (ii) ~10 mg of 4-methyl-*N*,*N*-di(prop-2-yn-1-yl)aniline dissolved in 0.7 mL of CDCl_3_, and (iii) a ~30 mmol equimolar mixture of oleic acid, linoleic acid, and the diruthenium compound **16a**, obtained by dissolving 5.08 mg of oleic acid, 5.05 mg of linoleic acid, and 24.21 mg of **16a** in 0.6 mL of CDCl_3_. The ^1^H and ^13^C 90° pulse lengths were 10 μs. Cholesteryl acetate: If not otherwise mentioned, all spectra were acquired with 64 k real data points with a relaxation delay of 2 s, with 128 scans for a total experimental time of approximately 7 min. The ^13^C spectral widths were 190 ppm, leading to an acquisition time of 1.71 s. Data were processed with 128 k data points using exponential multiplication with a line broadening of 1 Hz. 4-methyl-*N*,*N*-di(prop-2-yn-1-yl)aniline: The spectra were acquired with 64 k data points with a relaxation delay of 4 s, with 64 scans for a total experimental time of approximately 6 min. The ^13^C spectral widths were 190 ppm, leading to an acquisition time of 1.71 s. Data were processed with 128 k data points using exponential multiplication with a line broadening of 1 Hz. Mixture oleic acid/linoleic acid/**16a**: BAPT spectra were acquired with 64 k real data points with a relaxation delay of 1 s, with 1024 scans for a total experimental time of approximately 21 min. The ^13^C spectral widths were 270 ppm, leading to an acquisition time of 1.21 s. Data were processed with 128 k data points using exponential multiplication with a line broadening of 1 Hz. The 2D ^1^H-^13^C HSQC data were acquired using 2048 data points and 128 t_1_ increments. Prior to Fourier transformation, zero-filling to 1024 points and linear prediction in F1 (256 points) and weighting with a cosine-squared function were applied in both dimensions. The experimental parameters were as follows: acquisition time: 0.21 s; spectral width of 12 ppm in F2 and of 190 ppm in F1; relaxation delay: 1 s; number of scans: NS = 4; total experimental time: 10 min. The 2D ^1^H-^13^C HMBC data were acquired using 4096 data points and 256 t_1_ increments. Prior to Fourier transformation, zero-filling to 1024 points and linear prediction in F1 (512 points) and weighting with a cosine function were applied in both dimensions. The experimental parameters were as follows: acquisition time: 0.42 s; spectral width of 12 ppm in F2 and of 220 ppm in F1; relaxation delay: 1 s; number of scans: NS = 8; total experimental time: 40 min.

### 5.2. Numerical Simulations

The numerical simulations were performed with Microsoft Excel^®^, Office 365 for Windows. Relaxation effects during both pulse sequences were not considered.

### 5.3. NMR Simulations

The simulations were performed with the BRUKER NMRSIM program for Windows (version 7.0 2022).

## Figures and Tables

**Table 1:** Observable in-phase ^13^C coherences C_y_ for Cq, CH, CH_2_, and CH_3_ spin systems immediately before acquisition as obtained with the APT, APTjc [24], and BAPT sequences, the experimental parameters of which were set to obtain APT-like spectra. Relaxation effects were not considered. Terms in bold occurred when the delays were not optimally adjusted, Δ*_i_* ≠ 1/(2*J_CH_).

**APT**	**Δ_1_ = 1/^1^*J*^1^_CH_**
**Cq**	−C_y_
**CH**	C_y_ cos(π*J*Δ_1_)
**CH_2_**	C_y_ cos^2^(π*J*Δ_1_)
**CH_3_**	C_y_ cos^3^(π*J*Δ_1_)
**APTjc**	Δ_1_ = 1/(2*^1^*J*^1^_CH_)
**Cq**	−C_y_
**CH**	Term 1: C_y_sin^2^(π*J*Δ_1_) **Term 2: −C_y_cos^3^(π*J*Δ_1_)**
**CH_2_**	Term 1: −C_y_sin^4^(π*J*Δ_1_) **Term 2: 2C_y_sin^2^(π*J*Δ_1_)cos^3^(π*J*Δ_1_)** **Term 3: −C_y_cos^6^(π*J*Δ_1_)**
**CH_3_**	Term 1: C_y_sin^6^(π*J*Δ_1_) **Term 2: −3C_y_sin^4^(π*J*Δ_1_)cos^3^(π*J*Δ_1_)** **Term 3: 3C_y_sin^2^(π*J*Δ_1_)cos^6^(π*J*Δ_1_)** **Term 4: −C_y_cos^9^(π*J*Δ_1_)**
**BAPT**	Δ_1_ = 1/(2*^1^*J*^1^_CH_), **Δ_2_ = 1/(^1^*J*^2^_CH_),** Δ_3_ = 1/(2*^1^*J*^3^_CH_)
**Cq**	−C_y_
**CH**	Term 1: C_y_sin(π*J*Δ_3_)sin(π*J*Δ_1_) **Term 2: −C_y_cos(π*J*Δ_3_)cos(π*J*Δ_2_)cos(π*J*Δ_1_)**
**CH_2_**	Term 1: −C_y_sin^2^(π*J*Δ_3_)sin^2^(π*J*Δ_1_) **Term 2: 0.5C_y_sin(2π*J*Δ_3_)cos(π*J*Δ_2_)sin(2π*J*Δ_1_)** **Term 3: −C_y_cos^2^(π*J*Δ_3_)cos^2^(π*J*Δ_2_)cos^2^(π*J*Δ_1_)**
**CH_3_**	Term 1: C_y_sin^3^(π*J*Δ_3_)sin^3^(π*J*Δ_1_) **Term 2: −3C_y_sin^2^(π*J*Δ_3_)cos(π*J*Δ_3_)cos(π*J*Δ_2_)cos(π*J*Δ_1_)sin^2^(π*J*Δ_1_)** **Term 3: 3C_y_sin(π*J*Δ_3_)cos^2^(π*J*Δ_3_)cos^2^(π*J*Δ_2_)cos^2^(π*J*Δ_1_)sin(π*J*Δ_1_)** **Term 4: −C_y_cos^3^(π*J*Δ_3_)cos^3^(π*J*Δ_2_)cos^3^(π*J*Δ_1_)**

**Table 2:** Observable in-phase ^13^C coherences for CH, CH_2_, and CH_3_ spin systems immediately before acquisition with the APT, APTjc, and BAPT sequences set for recording Cq-only spectra.

**APT**	Δ_1_ = 1/(2*^1^*J*^1^_CH_)
**Cq**	−C_y_
**CH**	C_y_ cos(π*J*Δ_1_)
**CH_2_**	C_y_ cos^2^(π*J*Δ_1_)
**CH_3_**	C_y_ cos^3^(π*J*Δ_1_)
APTjc *	Δ_1_ = 1/(2*^1^*J*^1^_CH_)
**Cq**	−C_y_
**CH**	−C_y_cos^3^(π*J*Δ_1_)
**CH_2_**	−C_y_cos^6^(π*J*Δ_1_)
**CH_3_**	−C_y_cos^9^(π*J*Δ_1_)
**BAPT**	** Δ ** **_1_** = 1/(2*^1^*J*^1^_CH_), Δ **_2_** ** = 1/(^1^*J*^2^_CH_), Δ** **_3_** = 1/(2*^1^*J*^3^_CH_)
**Cq**	−C_y_
**CH**	−C_y_cos(π*J*Δ_3_)cos(π*J*Δ_2_)cos(π*J*Δ_1_)
**CH_2_**	−C_y_cos^2^(π*J*Δ_3_)cos^2^(π*J*Δ_2_)cos^2^(π*J*Δ_1_)
**CH_3_**	−C_y_cos^3^(π*J*Δ_3_)cos^3^(π*J*Δ_2_)cos^3^(π*J*Δ_1_)

* The APTjc pulse sequence, which is intended exclusively for measuring APT spectra [24], can also be used for measuring Cq-only (Figure S1).

## Data Availability

Data are contained within the article and Supplementary Materials.

## Supplementary Materials

The following supporting information can be downloaded at https://www.mdpi.com/article/10.3390/molecules29040809/s1, Figure S1: Pulse sequence of the modified CAPT3 for recording Cq-only spectra, Figure S2: Theoretical intensity of CH groups, APT and APTjc, 130–180 Hz, Figure S3: Theoretical intensity of CH groups, APTjc and BAPT, 125–170 Hz, Figure S4: Theoretical intensity of CH2 groups, APTjc and BAPT, 125–165 Hz, Figure S5: Theoretical intensity of CH3 groups, APTjc and BAPT, 110–135 Hz, Figure S6: Simulated Cq-only spectra with noise, APT, APTjc, and BAPT, 115–165 Hz, Figure S7: Theoretical intensity of CH groups, APTjc and BAPT, 130–250 Hz, Figure S8: Theoretical intensity of CH_2_ groups, APTjc and BAPT, 120–185 Hz, Figure S9: Theoretical intensity of CH_3_ groups, APTjc and BAPT, 110–135 Hz, Figure S10: Cq-only. Theoretical intensity of CH groups, APTjc and BAPT, 130–185 Hz, Figure S11: Simulated Cq-only spectra with noise, APT, APTjc, and BAPT, 115–165 Hz, Figure S12: Cq-only. Theoretical intensity of CH groups, APTjc and BAPT, 130–250 Hz, Figure S13: Simulated Cq-only spectra with noise, APT, APTjc, and BAPT, 115–250 Hz, Figure S14: Experimental APT, APTjc, and BAPT spectra of cholesteryl acetate, Figure S15: Experimental BAPT spectra of 4-methyl-*N*,*N*-di(prop-2-yn-1-yl)aniline, Figure S16: 2D HMBC and BAPT spectra of the mixture oleic acid, linoleic acid, and diruthenium compound **16a**, BAPT pulse program.
