# Supplementary material for: A Versatile Broadband Attached Proton Test Experiment for Routine 13C Nuclear Magnetic Resonance Spectroscopy
Source: Molecules. 2024 Feb 9;29(4):809. doi: 10.3390/molecules29040809 (PMC10893545; doi:10.3390/molecules29040809)
Supplement: Supplementary file 1 [file molecules-29-00809-s001.zip › molecules-2805176-supplementary.pdf]

A Versatile Broadband Attached Proton Test Experiment for Routine ^{13}C Nuclear Magnetic Resonance Spectroscopy

P. Bigler*, I. Gjuroski, D. Chakif, J. Furrer*

Department of Chemistry, Biochemistry and Pharmaceutical Sciences, University of Bern, Freiestrasse 3, 3012 Bern, Switzerland.

Supplementary Materials

Contents

	Page
**Figure S1** Pulse sequence of the modified CAPT3 for recording Cq-only spectra	**3**
**Figure S2** Theoretical intensity of CH groups, APT & APTjc, 130 – 180 Hz	**4**
**Figure S3** Theoretical intensity of CH groups, APTjc & BAPT, 125 – 170 Hz	**5**
**Figure S4** Theoretical intensity of CH_2 groups, APTjc & BAPT, 125 – 165 Hz	**6**
**Figure S5** Theoretical intensity of CH_3 groups, APTjc & BAPT, 110 – 135 Hz	**7**
**Figure S6** Simulated Cq-only spectra with noise, APT, APTjc & BAPT, 115 – 165 Hz	**8**
**Figure S7** Theoretical intensity of CH groups, APTjc & BAPT, 130 – 250 Hz	**9**
**Figure S8** Theoretical intensity of CH_2 groups, APTjc & BAPT, 120 – 185 Hz	**10**
**Figure S9** Theoretical intensity of CH_3 groups, APTjc & BAPT, 110 – 135 Hz	**11**
**Figure S10** Cq-only. Theoretical intensity of CH groups, APTjc & BAPT, 130 – 185 Hz	**12**
**Figure S11** Simulated Cq-only spectra with noise, APT, APTjc & BAPT, 115 – 165 Hz	**13**
**Figure S12** Cq-only. Theoretical intensity of CH groups, APTjc & BAPT, 130 – 250 Hz	**14**
**Figure S13** Simulated Cq-only spectra with noise, APT, APTjc & BAPT, 115 – 250 Hz	**15**
**Figure S14** Experimental APT, APTjc, and BAPT spectra of Cholesteryl acetate	**16**
**Figure S15** Experimental BAPT spectra of 4-methyl-*N,N*-di(prop-2-yn-1-yl)aniline	**17**
**Figure S16** 2D HMBC and BAPT spectra of the mixture oleic acid, linolenic acid, and diruthenium compound **16a**.	**18**
BAPT pulseprogram	**19**

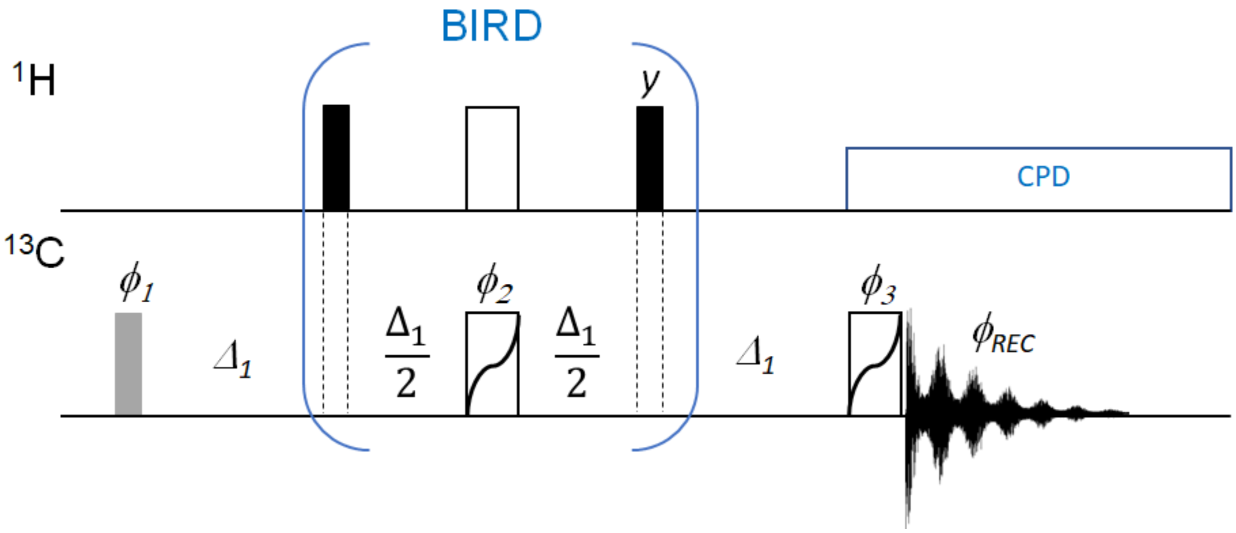

Figure S1. Pulse sequence of the modified CAPT3 for recording Cq-only spectra. Thin bars stand for 90° pulses, thick bars for 180° pulses. All ^{13}C 180° pulses can be replaced by broadband refocusing pulses. The first ^{13}C pulse may be set shorter than 90° to allow for a faster repetition rate (shown in grey). Δ_1 is set to an average value $1/(2\,^1J^1{}_{CH})$. The following phase cycling is applied ϕ_1 = *4(x), 4(-x), 4(y), 4(-y)*, ϕ_2 = *x, y, -x, -y, y, -x, -y, x, -x, -y, x, y, -y, x, y, -x*, ϕ_3 = *x, y, y, x,(y, x, x, y)$_2$, x, y, y, x*, ϕ_{REC} = *x, x, -x, -x, y, y, -y, -y.* Phases not shown are applied along the *x*-axis.

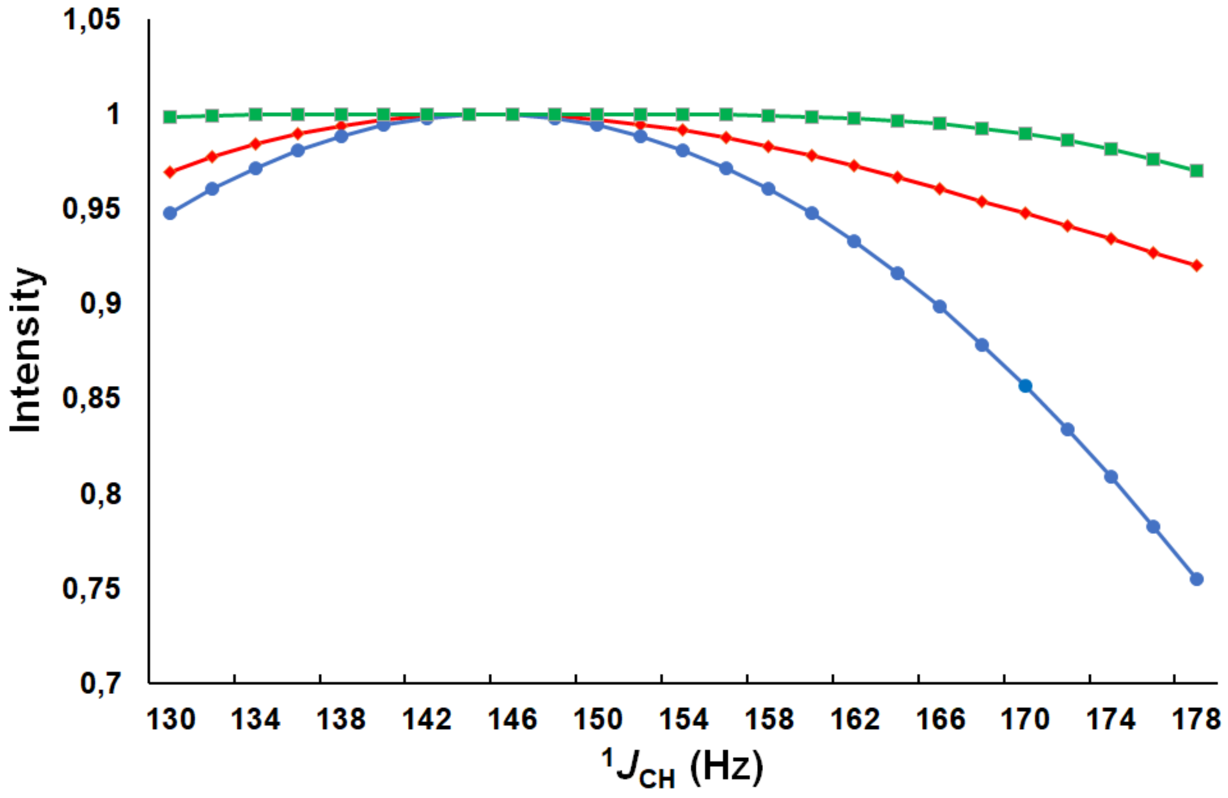

Figure S2. Theoretical intensity of CH groups as a function of the $^1J_{CH}$ coupling constant value for the APT (--), the APTjc with $\Delta_1 = 1/(2{*}^1J^1{}_{CH})$ during the BIRD sandwich (--), and the APTjc experiment with $\Delta_1 = 1/^1J^1{}_{CH}$ during the BIRD sandwich (--). Δ_1 was set to match a coupling constant value of 145 Hz. $^1J_{CH}$ coupling constant range: 130 – 180 Hz.

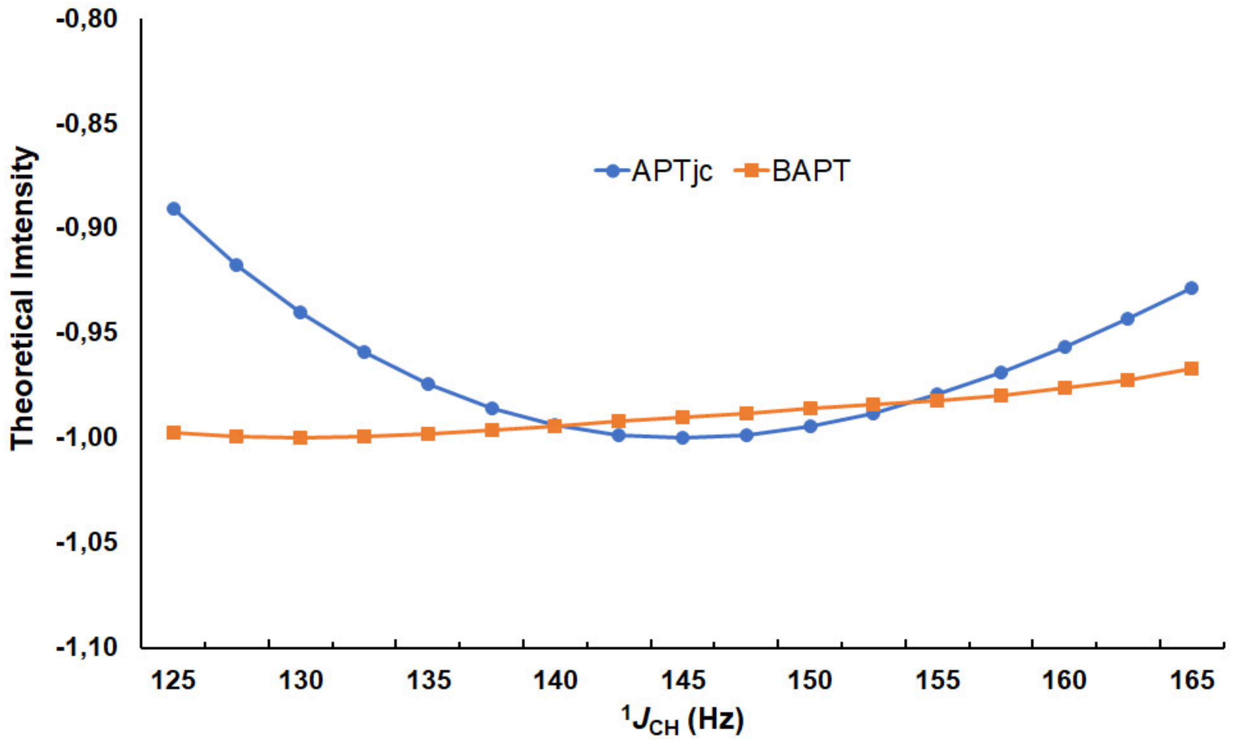

Figure S3. Theoretical intensity of CH groups as a function of the $^{1}J_{CH}$ coupling constant value for the APTjc pulse sequence, Δ_1 = 1/(2*$^{1}J^{1}_{CH}$) (--), and for the BAPT pulse sequence, Δ_2 = 1/$^{1}J^{2}_{CH}$ during the BIRD sandwich (--). For APTjc, Δ_1 was set to match a coupling constant value of 145 Hz. For BAPT, Δ_1 and Δ_3 were set to match a coupling constant value of 130 Hz, and Δ_2 was set to match a coupling constant value of 175 Hz. Equations provided in table 1 were used for the simulations.

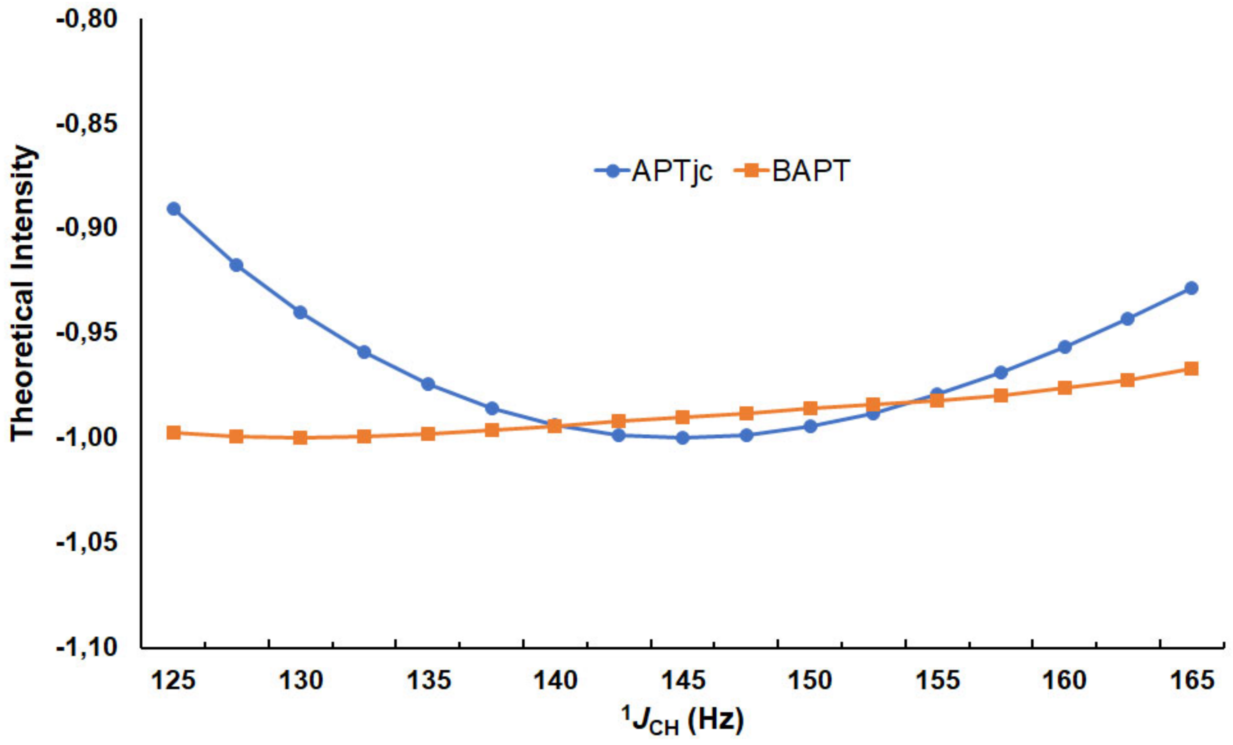

Figure S4. Theoretical intensity of CH_2 groups as a function of the ${}^{1}J_{CH}$ coupling constant value for the APTjc pulse sequence, $\Delta_1 = 1/(2{*}{}^{1}J^{1}{}_{CH})$ (--), and for the BAPT pulse sequence, $\Delta_2 = 1/{}^{1}J^{2}{}_{CH}$ during the BIRD sandwich (--). For APTjc, Δ_1 was set to match a coupling constant value of 145 Hz. For BAPT, Δ_1 and Δ_3 were set to match a coupling constant value of 130 Hz, and Δ_2 was set to match a coupling constant value of 175 Hz. Equations provided in table 1 were used for the simulations.

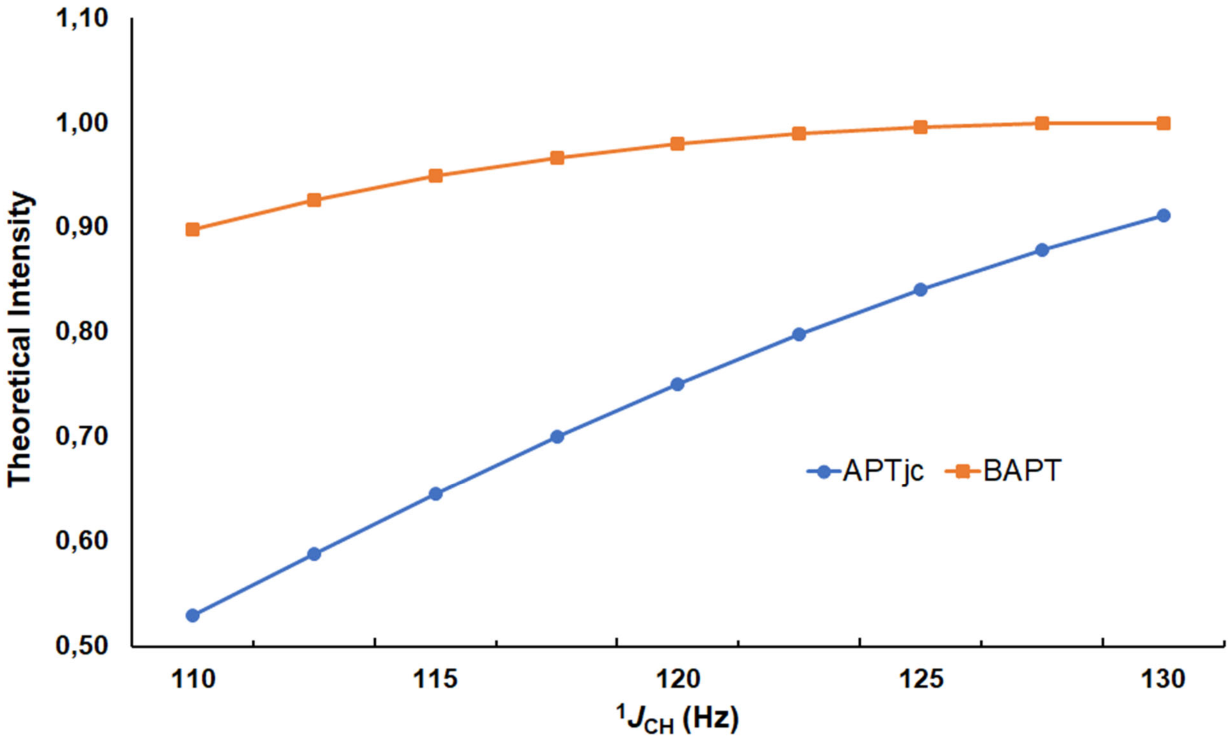

Figure S5. Theoretical intensity of CH_3 groups as a function of the $^1J_{CH}$ coupling constant value for the APTjc pulse sequence, $\Delta_1 = 1/(2{*}^1J^1_{CH})$ (--), and for the BAPT pulse sequence, $\Delta_2 = 1/^1J^2_{CH}$ during the BIRD sandwich (--). For APTjc, Δ_1 was set to match a coupling constant value of 145 Hz. For BAPT, Δ_1 and Δ_3 were set to match a coupling constant value of 130 Hz, and Δ_2 was set to match a coupling constant value of 175 Hz. Equations provided in table 1 were used for the simulations.

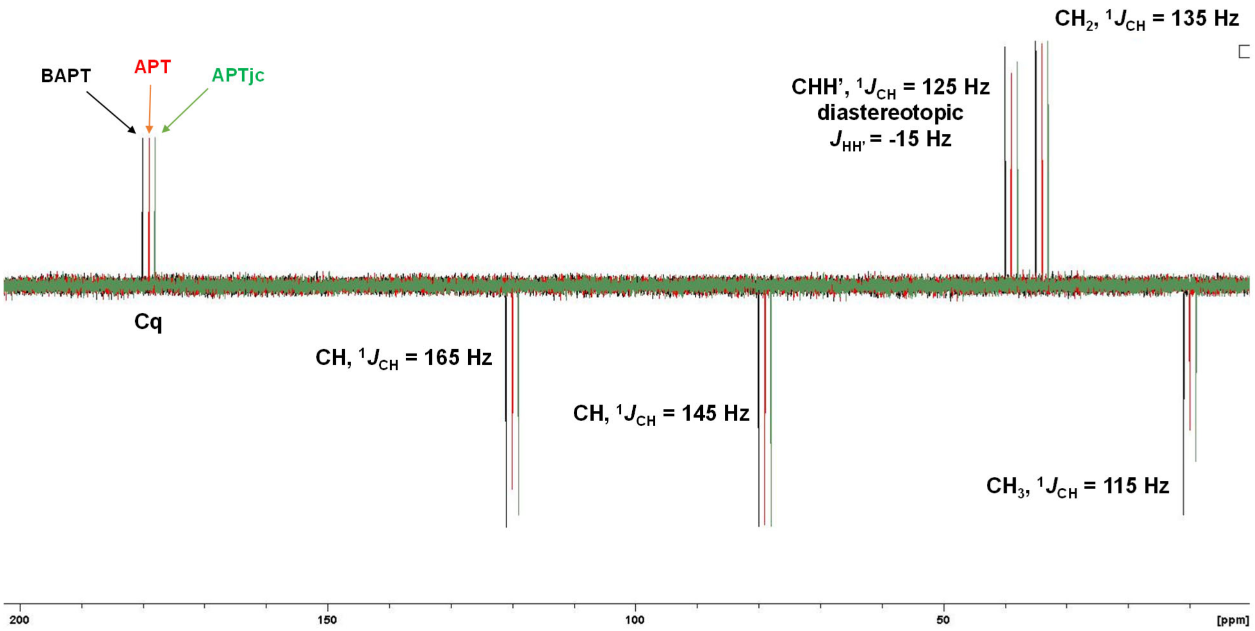

Figure S6. Simulated spectra using the APT (--), the APTjc, $\Delta_1 = 1/(2*^1J^1_{CH})$ (--) and the BAPT pulse sequences, $\Delta_2 = 1/^1J^2_{CH}$ during the BIRD sandwich (--). For APT and APTjc, Δ_1 was set to match a coupling constant value of 145 Hz. For BAPT, Δ_1 and Δ_3 were set to match a coupling constant value of 130 Hz, and Δ_2 was set to match a coupling constant value of 175 Hz. The spin system used for simulation is: quaternary carbon, δ = 180 ppm, T_1 = 5s, T_2 = 1s; aromatic CH carbon, δ = 120 ppm, T_1 = 2s, T_2 = 1s, $^1J_{CH}$ = 165 Hz; alkoxy CH carbon, δ = 80 ppm, T_1 = 2s, T_2 = 1s, $^1J_{CH}$ = 145 Hz; aliphatic diastereotopic CHH' carbon, δ = 40 ppm, T_1 = 2s, T_2 = 1s, $^1J_{CH}$ = 135 Hz, $J_{HH'}$ = -15 Hz; aliphatic CH_2 carbon, δ = 35 ppm, T_1 = 2s, T_2 = 1s, $^1J_{CH}$ = 125 Hz, aliphatic CH_3 carbon, δ = 10 ppm, T_1 = 3s, T_2 = 1s, $^1J_{CH}$ = 115 Hz. Full relaxation was considered and random noise has been added. Simulations were performed using NMRSIM for Windows. Acquisition parameters: Spectrometer frequency: 400 MHz, 64 scans, relaxation delay: 2s, 90° pulse length 5 μs, S/N: 50.

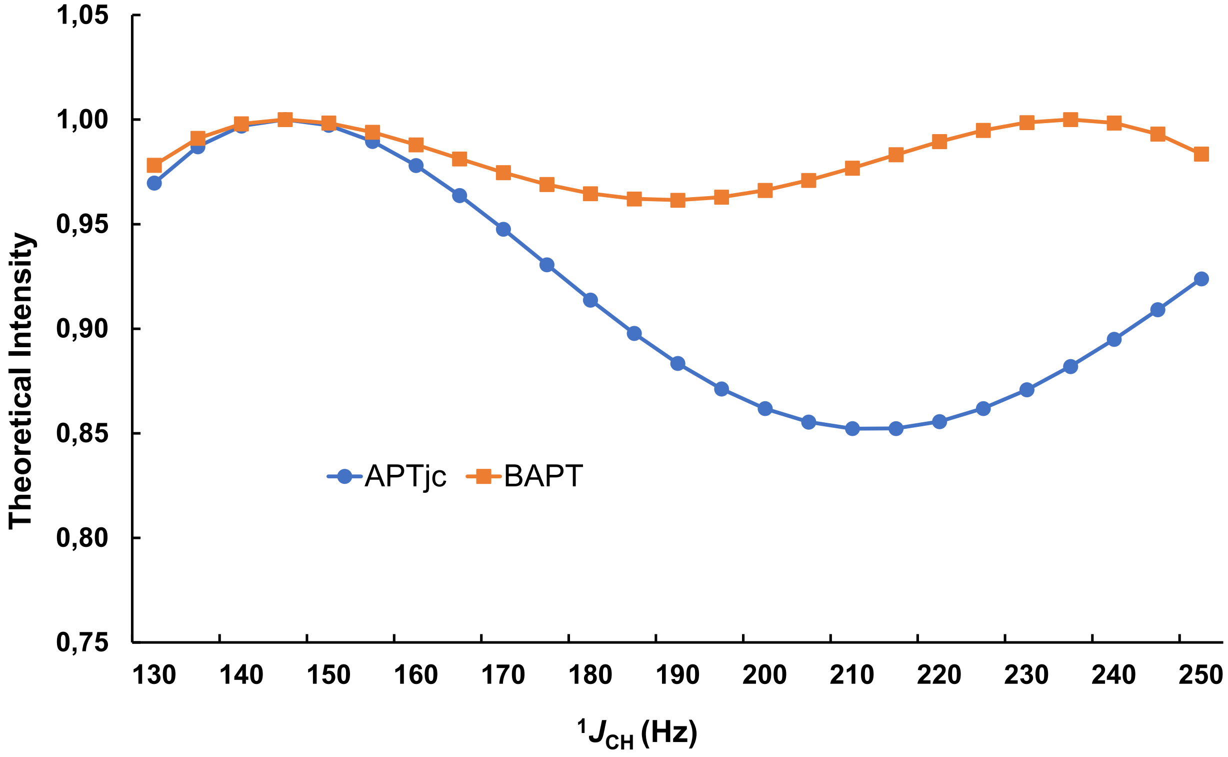

Figure S7. Theoretical intensity of CH groups as a function of the $^1J_{CH}$ coupling constant value for the APTjc pulse sequence, $\Delta_1 = 1/(2{*}^1J^1_{CH})$ (--), and for the BAPT pulse sequence, $\Delta_2 = 1/^1J^2_{CH}$ during the BIRD sandwich (--). For APTjc, Δ_1 was set to match a coupling constant value of 145 Hz. For BAPT, Δ_1 and Δ_3 were set to match a coupling constant value of 145 Hz, and Δ_2 was set to match a coupling constant value of 235 Hz. Equations provided in table 1 were used for the simulations.

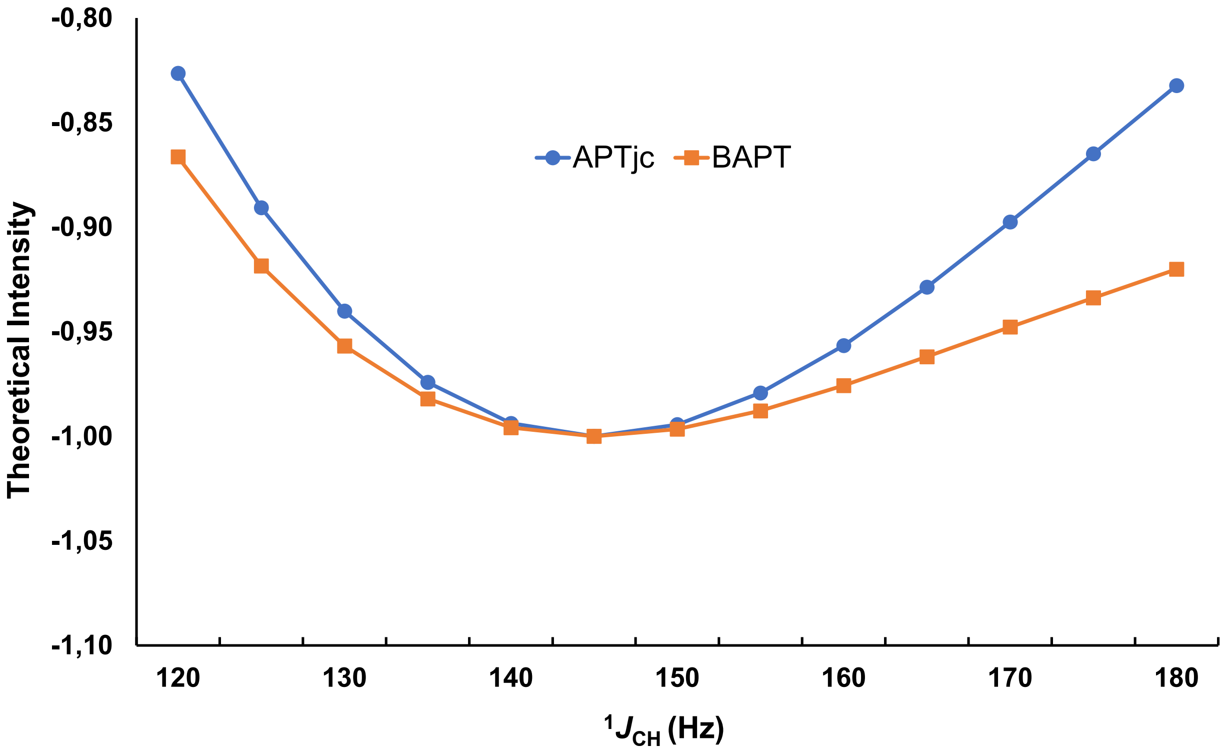

Figure S8. Theoretical intensity of CH_2 groups as a function of the $^1J_{CH}$ coupling constant value for the APTjc pulse sequence, $\Delta_1 = 1/(2^{*1}J^1_{CH})$ (--), and for the BAPT pulse sequence, $\Delta_2 = 1/^1J^2_{CH}$ during the BIRD sandwich (--). For APTjc, Δ_1 was set to match a coupling constant value of 145 Hz. For BAPT, Δ_1 and Δ_3 were set to match a coupling constant value of 145 Hz, and Δ_2 was set to match a coupling constant value of 235 Hz. Equations provided in table 1 were used for the simulations.

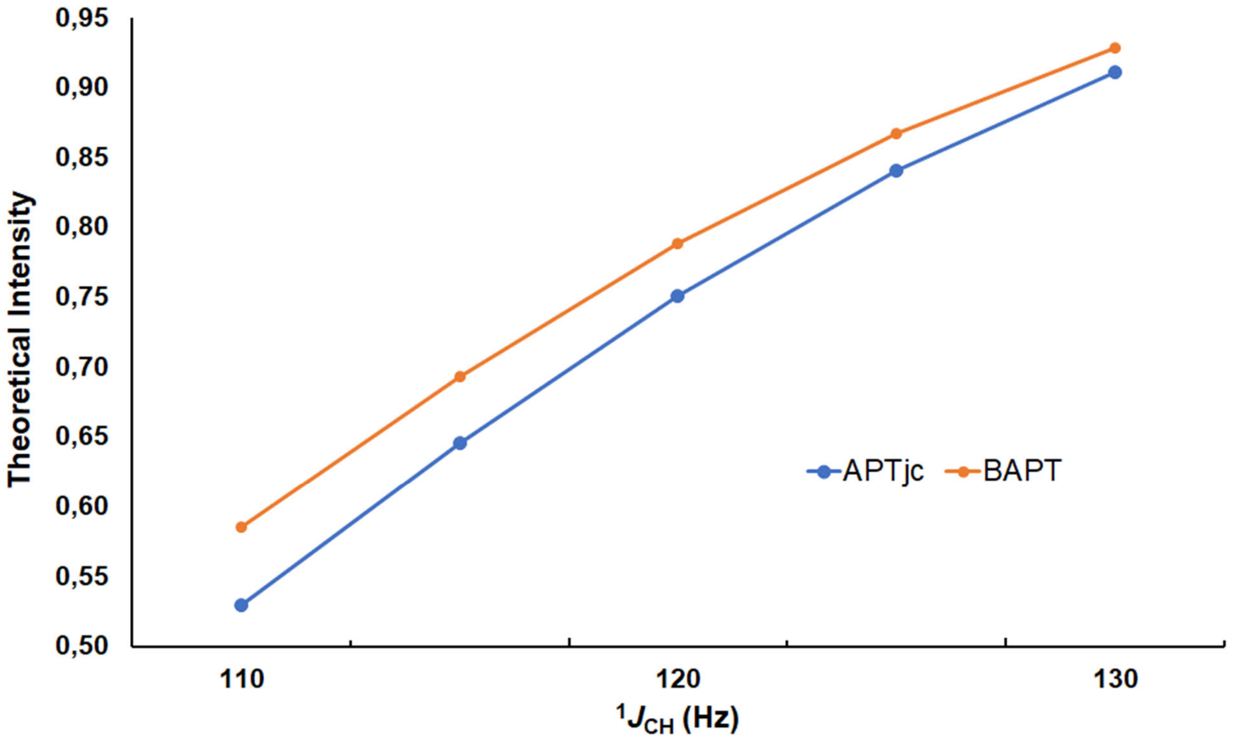

Figure S9. Theoretical intensity of CH_3 groups as a function of the $^1J_{CH}$ coupling constant value for the APTjc pulse sequence, $\Delta_1 = 1/(2{*}^1J^1_{CH})$ (--), and for the BAPT pulse sequence, $\Delta_2 = 1/^1J^2_{CH}$ during the BIRD sandwich (--). For APTjc, Δ_1 was set to match a coupling constant value of 145 Hz. For BAPT, Δ_1 and Δ_3 were set to match a coupling constant value of 145 Hz, and Δ_2 was set to match a coupling constant value of 235 Hz. Equations provided in table 1 were used for the simulations.

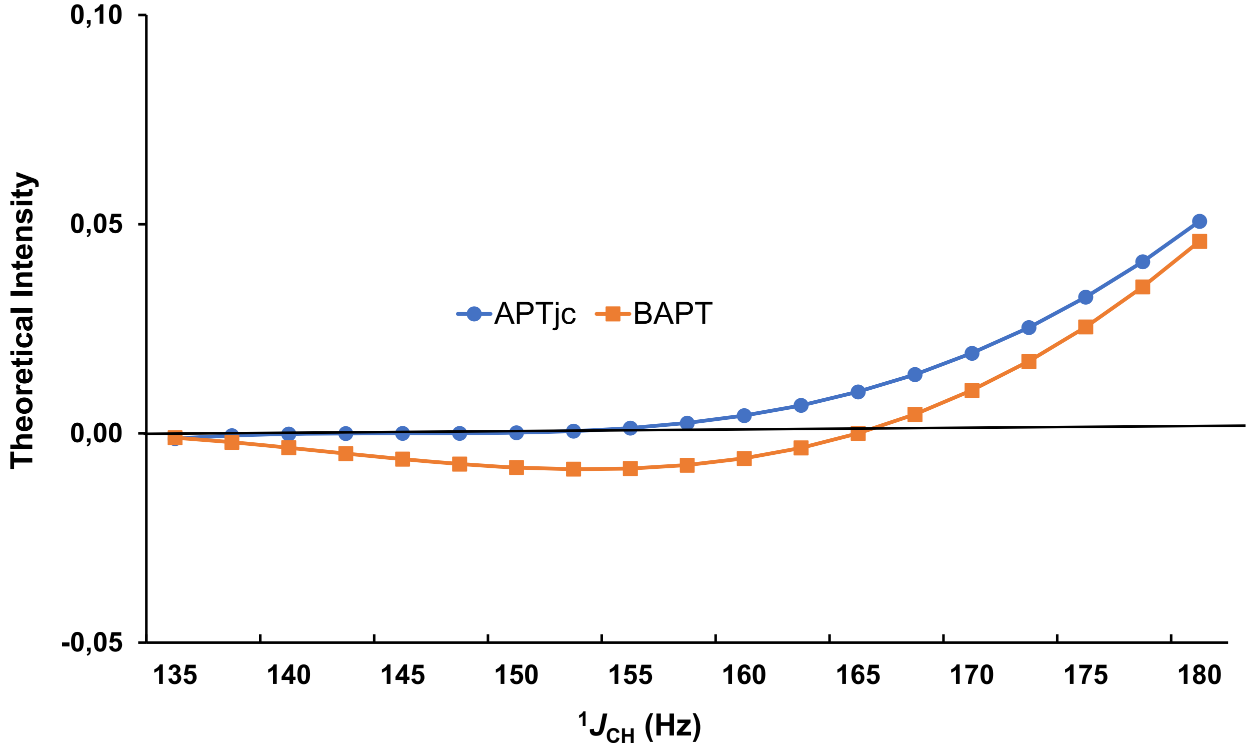

Figure S10. Cq-only mode. Theoretical residual intensity of CH groups as a function of the $^{1}J_{CH}$ coupling constant value for the modified APTjc pulse sequence, Δ_1 = 1/(2*$^{1}J^{1}_{CH}$) (--) and for the BAPT pulse sequence, Δ_2 = 1/(2*$^{1}J^{2}_{CH}$) during the BIRD sandwich (--). For APTjc, Δ_1 was set to match a coupling constant value of 145 Hz. For BAPT, Δ_1 and Δ_3 were set to match a coupling constant value of 130 Hz, and Δ_2 was set to match a coupling constant value of 165 Hz. Equations provided in table 2 were used for the simulations.

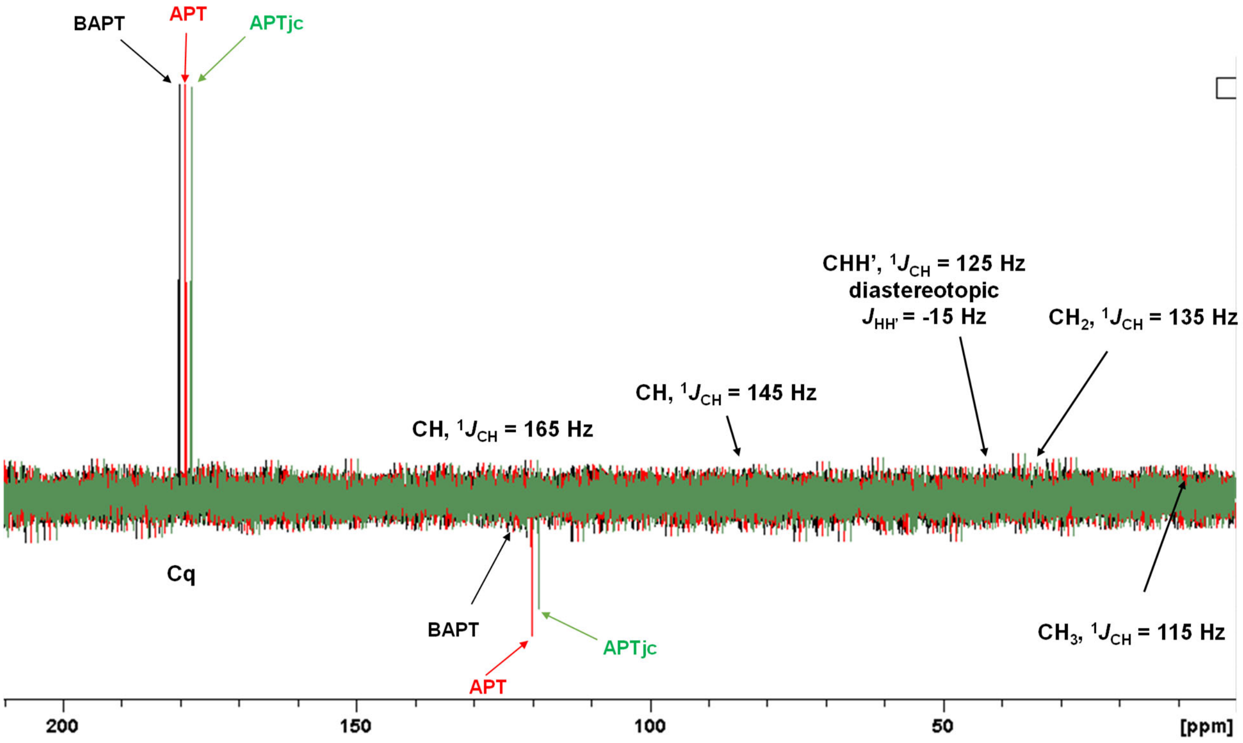

Figure S11. Simulated Cq-only spectra for the APT pulse sequence (--), the modified APTjc pulse sequence, $\Delta_1 = 1/(2^{*1J1CH})$ (--) and the BAPT pulse sequence, $\Delta_2 = 1/^1J^2{}_{CH}$ during the BIRD sandwich (--). The spin system and all parameters are identical to those used for Figure S6.

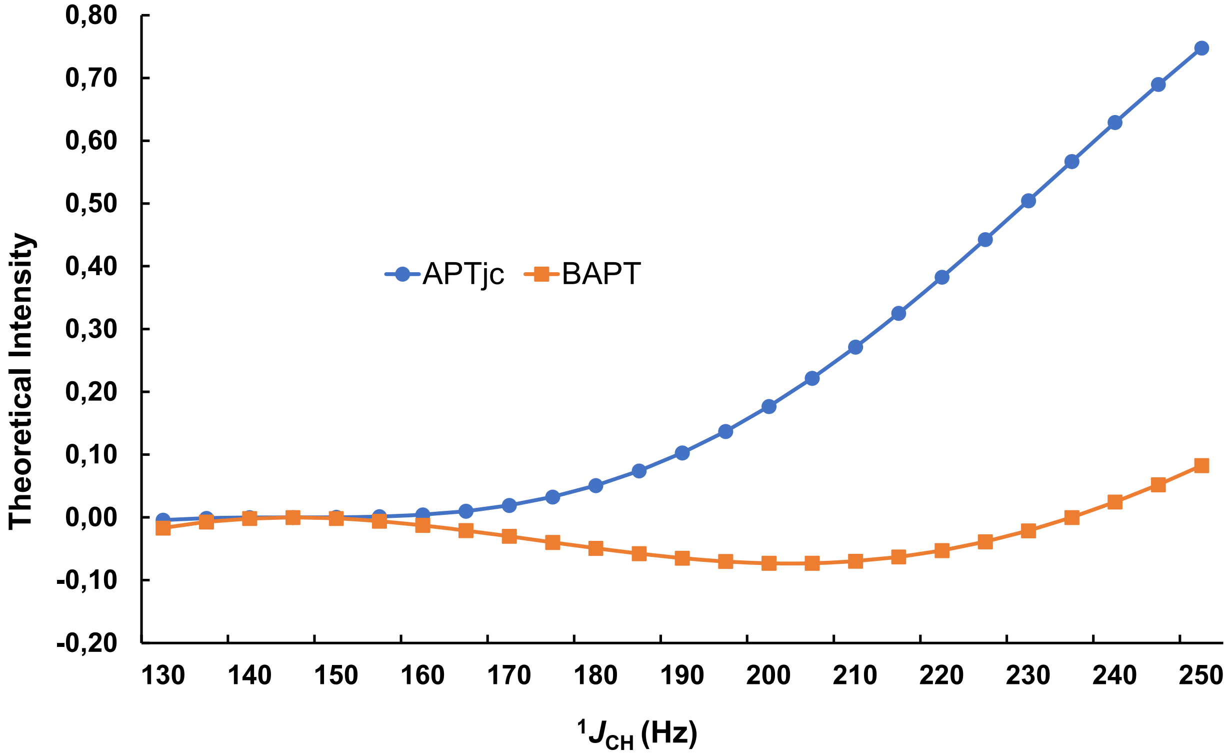

Figure S12. Cq-only mode. Theoretical residual intensity of CH groups as a function of the $^1J_{CH}$ coupling constant value for the modified APTjc pulse sequence, Δ_1 = 1/(2*$^1J^1{}_{CH}$) (--) and for the BAPT pulse sequence, Δ_2 = 1/$^1J^2{}_{CH}$ during the BIRD sandwich (--). For APTjc, Δ_1 was set to match a coupling constant value of 145 Hz. For BAPT, Δ_1 and Δ_3 were set to match a coupling constant value of 145 Hz, and Δ_2 was set to match a coupling constant value of 235 Hz. Equations provided in table 2 were used for the simulations.

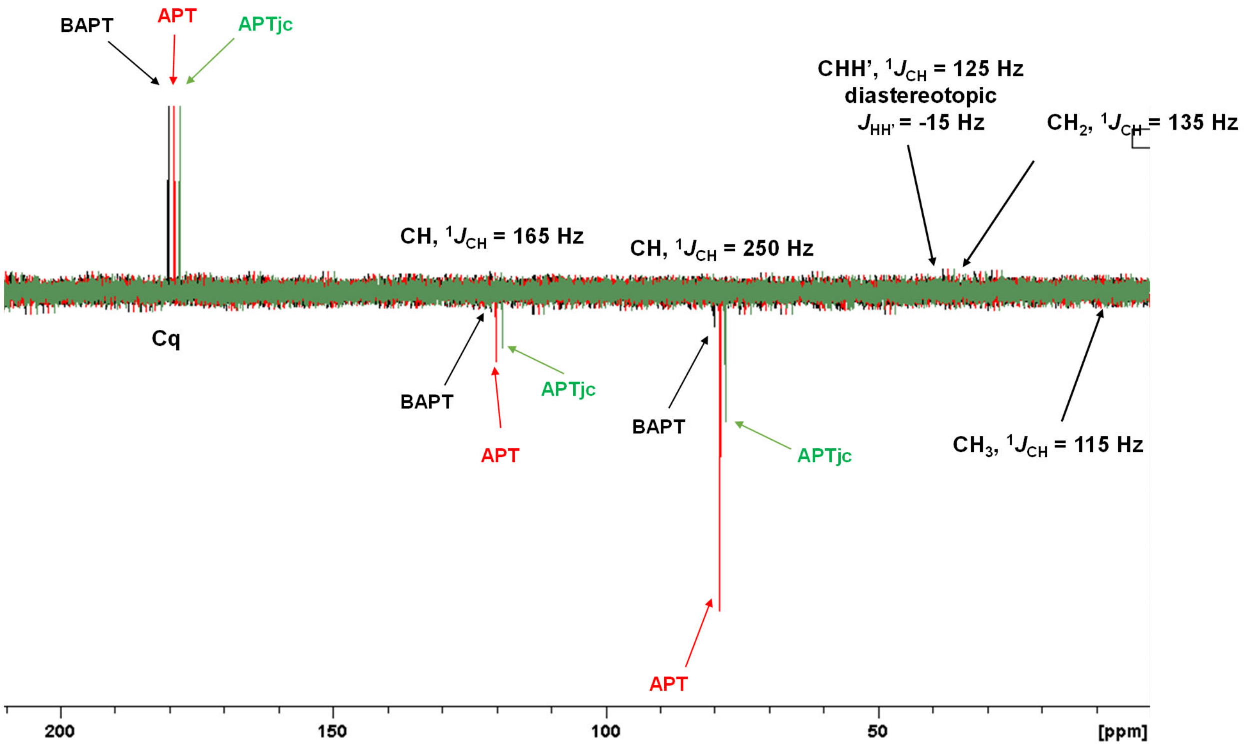

Figure S13. Simulated Cq-only spectra for the APT pulse sequence (--), the modified APTjc pulse sequence, $\Delta_1 = 1/(2{*}^1J^1{}_{CH})$ (--) and the BAPT pulse sequence, $\Delta_2 = 1/^1J^2{}_{CH}$ during the BIRD sandwich (--). For APT and APTjc, Δ_1 was set to match a coupling constant value of 145 Hz. For BAPT, Δ_1 and Δ_3 were set to match a coupling constant value of 145 Hz, and Δ_2 was set to match a coupling constant value of 240 Hz. The spin system used for simulation is: quaternary carbon, δ = 180 ppm, T_1 = 5s, T_2 = 1s; aromatic CH carbon, δ = 120 ppm, T_1 = 2s, T_2 = 1s, $^1J_{CH}$ = 165 Hz; alkyne CH carbon, δ = 80 ppm, T_1 = 3s, T_2 = 1s, $^1J_{CH}$ = 250 Hz; aliphatic diastereotopic CHH' carbon, δ = 40 ppm, T_1 = 2s, T_2 = 1s, $^1J_{CH}$ = 135 Hz, $J_{HH'}$ = -15 Hz; aliphatic CH_2 carbon, δ = 35 ppm, T_1 = 2s, T_2 = 1s, $^1J_{CH}$ = 125 Hz, aliphatic CH_3 carbon, δ = 10 ppm, T_1 = 3s, T_2 = 1s, $^1J_{CH}$ = 115 Hz. Full relaxation was considered and random noise has been added. Simulations were performed using NMRSIM for Windows. Acquisition parameters: Spectrometer frequency: 400 MHz, 64 scans, relaxation delay: 2s, 90° pulse length 5 μs, S/N: 50.

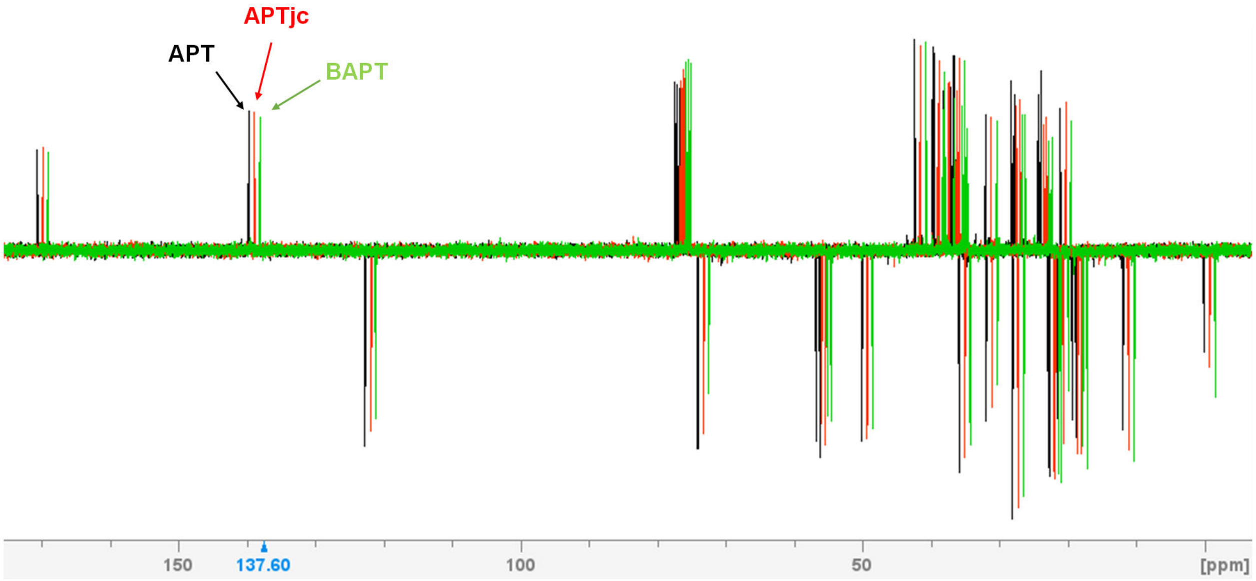

Figure S14. Superimposed APT (black), APTjc (red), and BAPT (green) spectra of cholesteryl acetate. For APT and APTjc, the delay Δ was adjusted to a coupling constant of 145 Hz, while for BAPT, Δ_1 and Δ_3 were adjusted to a coupling constant of 130 Hz, and Δ_2 was adjusted to a coupling constant of 170 Hz.

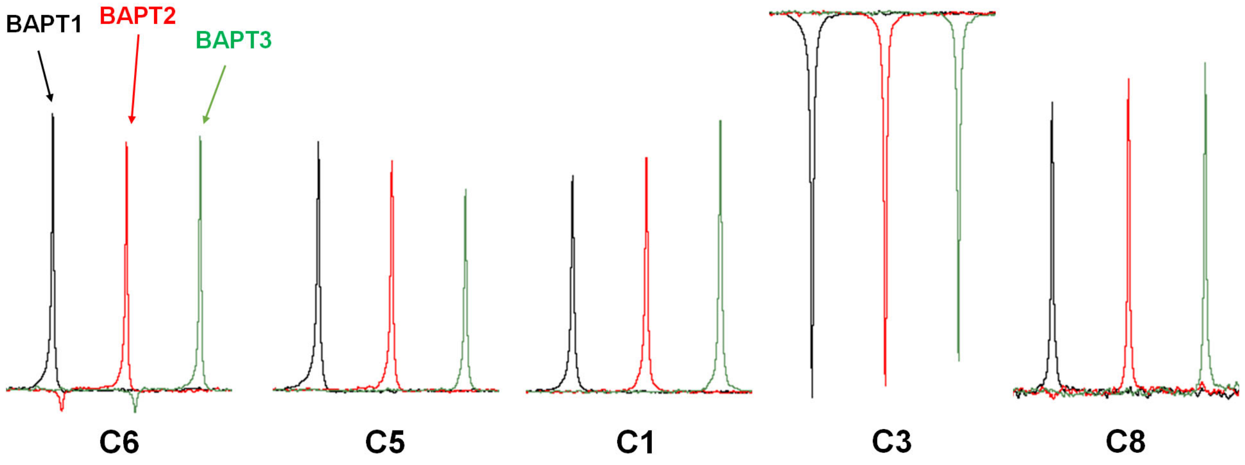

Figure S15. Resonances of C1, C3, C5, C6, and C8 of ~10 mg of 4-methyl-*N,N*-di(prop-2-yn-1-yl)aniline dissolved in 0.7 mL $CDCl_3$ recorded with the BAPT experiments for 3 different optimizations. Black: the delays Δ_1 *and* Δ_3 were adjusted for a coupling constant $^1J^1_{CH}$ of 145 Hz, and the delay Δ_2 for a coupling constant $^1J^2_{CH}$ of 240 Hz. Red: the delays Δ_1 *and* Δ_3 were adjusted for a coupling constant $^1J^{1,3}_{CH}$ of 130 Hz, and the delay Δ_2 for a coupling constant $^1J^2_{CH}$ of 240 Hz. Green: the delays Δ_1 *and* Δ_3 were adjusted for a coupling constant $^1J^{1,3}_{CH}$ of 125 Hz, and the delay Δ_2 for a coupling constant $^1J^2_{CH}$ of 220 Hz.

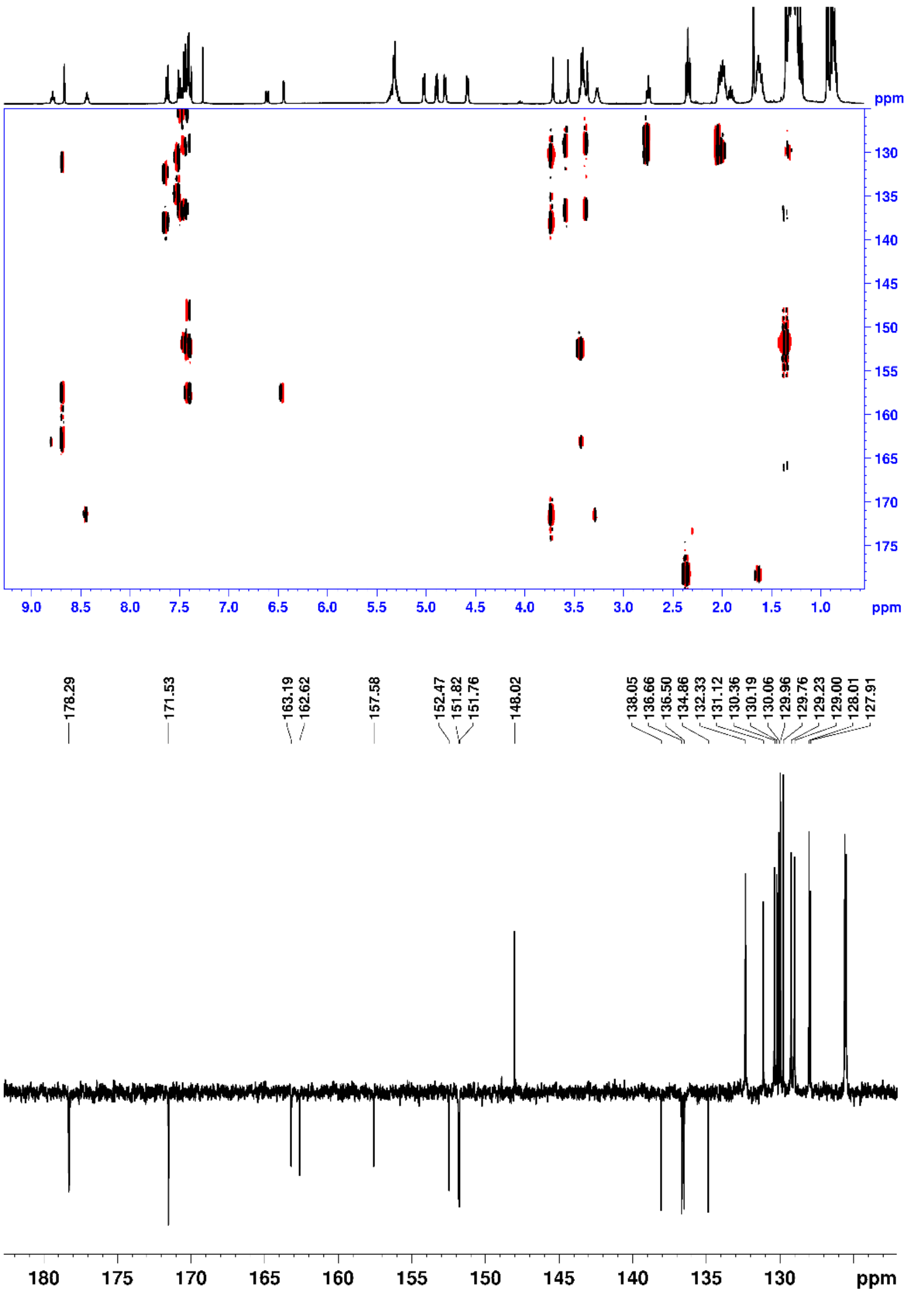

Figure S16. Top: Part of the 7 Hz optimized 2D HMBC spectrum of the equimolar mixture of oleic acid, linolenic acid and compound 16a (30 mmol of each compound dissolved in $CDCl_3$). Bottom: BAPT spectrum of the same mixture. The experimental parameters of the BAPT experiments were identical to those used for Figure 9.

```
;bapt
;avance-version (02/05/31)
;broadband attached proton test
;
;P Bigler, J Furrer, 2023
;
#include <Avance.incl>

"p2=p1*2"
"p4=p3*2"
"p13=(cnst11-1)*p3"
"p14=(2-cnst11)*p3"
"d2=1/(2*cnst2)"
"d3=1/(2*cnst3)"
"d20=(2-cnst11)*d3+(cnst11-1)*d3/2"
"d23=(cnst11-1)*(d2-(1/(2*cnst4)))"
"d22=d2-(cnst11-1)*d23"
"d11=30m"

1 ze
  d11 pl12:f2
  d11 cpd2:f2
2 30m
  d1 pl1:f1
  4u do:f2
  p0 ph1
  d2 pl2:f2
  (p13 ph5):f2                          ;Cq only
  (p14 ph6):f2                          ;APT

  d20                              ;APT: 1/(2*J(CH)) Cq only: 1/(4*J(CH))
  (p2 ph2) (p4 ph3):f2
  d20                                   ;APT: 1/(2*J(CH)) Cq only: 1/(4*J(CH))
```

```
  (p13 ph7):f2                          ;Cq only
  (p14 ph8):f2                          ;APT

  d22 pl12:f2
  d23 cpd2:f2
  d21
  p2 ph4
  d21
  go=2 ph31
  30m mc #0 to 2 F0(zd)
  d11 do:f2
exit

ph1=0 0 0 0 1 1 1 1 2 2 2 2 3 3 3 3
ph2=0 1 2 3 1 2 3 0 2 3 0 1 3 0 1 2
ph3=0
ph4=0 1 1 0 1 0 0 1 1 0 0 1 0 1 1 0
ph5=0 0 2 2
ph6=0
ph7=1 3
ph8=2
ph31=0 0 2 2 1 1 3 3

;pl1 : f1 channel - power level for pulse (default)
;pl12: f2 channel - power level for CPD/BB decoupling

;p0 : f1 channel - for any flip angle
;p1 : f1 channel - 90 degree high power pulse
;p3 : f2 channel - 90 degree high power pulse

;d1 : relaxation delay; 1-5 * T1
;d11: delay for disk I/O                [30 msec]

;d2 : 1/(2J(XH)          according to cnst2
;d3 : 1/(2J(XH)          according to cnst3
;d21: delay for second echo             [100 usec to 1msec]

;cnst2: ~130 Hz
;cnst3: ~180 Hz (BIRD)
```

;cnst4: ~130 Hz, or cnst4 > cnst2 (for Cq-only)
;cnst11: = 1 X, XH2 positive, XH, XH3 negative
;   2 X only
;ns : 4 * n, total number of scans: NS * TD0
;ds : 8
;cpd2: decoupling according to sequence defined by cpdprg2
;pcpd2: f2 channel - 90 degree pulse for decoupling sequence
